# Functional Trachea Reconstruction Using 3D‐Bioprinted Native‐Like Tissue Architecture Based on Designable Tissue‐Specific Bioinks

**DOI:** 10.1002/advs.202202181

**Published:** 2022-07-26

**Authors:** Yingying Huo, Yong Xu, Xiaodi Wu, Erji Gao, Anqi Zhan, Yujie Chen, Yixin Zhang, Yujie Hua, Wojciech Swieszkowski, Yu Shrike Zhang, Guangdong Zhou

**Affiliations:** ^1^ Department of Plastic and Reconstructive Surgery Shanghai 9th People's Hospital Shanghai Jiao Tong University School of Medicine Shanghai Key Laboratory of Tissue Engineering Shanghai 200011 P. R. China; ^2^ National Tissue Engineering Center of China Shanghai 200241 P. R. China; ^3^ Department of Thoracic Surgery Shanghai Pulmonary Hospital School of Medicine Tongji University Shanghai 200433 P. R. China; ^4^ Research Institute of Plastic Surgery Weifang Medical University Weifang Shandong 261053 P. R. China; ^5^ Uli Schwarz Quantitative Biology Core Facility Bio‐Med Big Data Center CAS Key Laboratory of Computational Biology Shanghai Institute of Nutrition and Health Chinese Academy of Sciences Shanghai 200031 P. R. China; ^6^ Materials Design Division Faculty of Materials Science and Engineering Warsaw University of Technology Warsaw 02‐507 Poland; ^7^ Division of Engineering in Medicine Department of Medicine Brigham and Women's Hospital Harvard Medical School Cambridge MA 02139 USA

**Keywords:** 3D bioprinting, cartilage regeneration, functional trachea reconstruction, tissue‐specific bioink, vascularization

## Abstract

Functional segmental trachea reconstruction remains a remarkable challenge in the clinic. To date, functional trachea regeneration with alternant cartilage‐fibrous tissue‐mimetic structure similar to that of the native trachea relying on the three‐dimensional (3D) bioprinting technology has seen very limited breakthrough. This fact is mostly due to the lack of tissue‐specific bioinks suitable for both cartilage and vascularized fibrous tissue regeneration, as well as the need for firm interfacial integration between stiff and soft tissues. Here, a novel strategy is developed for 3D bioprinting of cartilage‐vascularized fibrous tissue‐integrated trachea (**CVFIT**), utilizing photocrosslinkable tissue‐specific bioinks. Both cartilage‐ and fibrous tissue‐specific bioinks created by this study provide suitable printability, favorable biocompatibility, and biomimetic microenvironments for chondrogenesis and vascularized fibrogenesis based on the multicomponent synergistic effect through the hybrid photoinitiated polymerization reaction. As such, the tubular analogs are successfully bioprinted and the ring‐to‐ring alternant structure is tightly integrated by the enhancement of interfacial bonding through the amidation reaction. The results from both the trachea regeneration and the in situ trachea reconstruction demonstrate the satisfactory tissue‐specific regeneration along with realization of mechanical and physiological functions. This study thus illustrates the 3D‐bioprinted native tissue‐like trachea as a promising alternative for clinical trachea reconstruction.

## Graphical abstract

This study reports the development of a novel strategy for three‐dimensional (3D) bioprinting of trachea based on designable tissue‐specific bioinks with chondrogenic and fibrogenic/angiogenic microenvironments. The 3D‐bioprinted trachea‐analogue successfully regenerated cartilage‐vascularized fibrous tissue‐alternant architecture with biomimetic mechanical and physiological characteristics close to those of the native trachea and realized in situ functional trachea reconstruction.

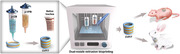

## 1 Introduction

Functional segmental trachea reconstruction has always been a grand challenge in the clinic because the key difficulty in the lack of ideal tracheal substitutes.^[^ ^1^, ^2^, ^3^ ^]^ The advancement of tissue engineering technologies has offered promising potentials to provide tracheal grafts with certain functions.^[^ ^4^, ^5^, ^6^, ^7^ ^]^ Currently, the regeneration using tissue‐engineered tubular cartilage constructs has been successfully achieved,^[^ ^8^, ^9^ ^]^ but such a tubular trachea configuration would inevitably cause mechanical rigidity, also lacking vascularization of connective tissues interspersed between the cartilages and epithelium on the tracheal liminal surface. Of note, the native trachea has a heterogeneous structure of alternant cartilage rings (**C** rings) and vascularized fibrous tissue rings (**VF** rings), as well as tracheal epithelium, which is essential to the realization of trachea's mechanical and physiological functions.^[^ ^10^ ^]^

To date, satisfactory trachea reconstruction with alternant tubular structures has seen limited clinical breakthrough due to the difficulties in heterogeneous tissue regeneration, structural mimicry, as well as integration of vascularization and epithelium. In our previous work, an innovative concept based on ring‐to‐tube assembly, via alternately stacking cartilage rings and polymer rings (cell‐free) to form a tubular structure, was reported to emulate the physiological structure of the native trachea.^[^ ^11^ ^]^ However, the following issues had not been resolved in this previous study: i) synthetic materials lacked full biocompatibility and tissue‐specific microenvironmental cues; ii) a long‐term in vitro culture was needed to achieve satisfactory cartilage formation; iii) manual preparation of alternant tubular structure was not precisely controllable; iv) connective tissue regeneration relied on random cell infiltration, leading to instable tissue formation and inferior vascularization; v) interfacial integration between **C** rings and **VF** rings were not rationally designed; and vi) systematic functional evaluations were not conducted in the regenerated trachea. The latest report based on the same strategy verified the feasibility of biomimetic trachea regeneration through replacing the polymer scaffolds by the natural scaffolds and replacing chondrocytes by bone marrow stem cells (BMSCs).^[^ ^12^ ^]^ Nevertheless, most of the above‐mentioned problems have not been addressed, and the use of BMSCs might increase the risk of ossification after long‐term implantation in the tracheal environment.

Emerging three‐dimensional (3D) extrusion bioprinting strategies^[^ ^13^, ^14^, ^15^, ^16^, ^17^, ^18^, ^19^, ^20^ ^]^ based on photocrosslinkable hydrogels^[^ ^21^, ^22^, ^23^, ^24^, ^25^, ^26^ ^]^ have proven to be an attractive technology for the construction of heterogeneous tissue architectures.^[^ ^27^, ^28^ ^]^ The cell‐laden photocrosslinkable bioinks facilitate the construction of biomimetic tissue equivalents for numerous biomedical applications, such as accurate shape‐control similar to actual tissues (e.g., ear, nose, and trachea), precise positioning of multiple types of cells and bioinks, and vascularization designs.^[^ ^29^, ^30^, ^31^ ^]^ Furthermore, the photocrosslinkable bioinks enable building free‐standing complex and volumetric architectures via rapid photocrosslinking mechanisms without any supporting materials in the case of vat‐polymerization bioprinting.^[^ ^32^ ^]^ Nevertheless, current 3D‐bioprinted trachea grafts have mostly focused on the production of local patches and have not been able to realize reliable cartilage regeneration.^[^ ^33^, ^34^, ^35^, ^36^ ^]^ Especially, functional trachea regeneration with alternant cartilage‐fibrous tissue‐mimetic structure similar to the native trachea, using the 3D bioprinting technology, has only seen very limited breakthrough, which is mainly attributed to the lack of tissue‐specific bioinks suitable for both cartilage and vascularized fibrous tissue regeneration. Additionally, the dual‐material extrusion bioprinting method based on layer‐by‐layer stacking when using hydrogels as bioinks oftentimes suffer from interfacial separation owing to the weak physical fusion instead of strong chemical bonding.^[^ ^37^, ^38^ ^]^ Therefore, there is an urgent need for developing tissue‐specific bioinks to simultaneously meet the following requirements in functional trachea bioprinting: i) good printability; ii) stable cell viability during bioprinting; iii) favorable biocompatibility after bioprinting; iv) tissue‐specific microenvironments suitable for alternant cartilage and vascularized fibrous tissue regeneration for supporting epithelialization of the regenerated trachea; and v) strong interfacial integration between alternant ring‐to‐ring structures. Because of the difficulties meeting all these requirements simultaneously, 3D bioprinting of trachea with an alternant cartilage‐vascularized fibrous tissue architecture remains challenging to realize.

**Figure 1:**
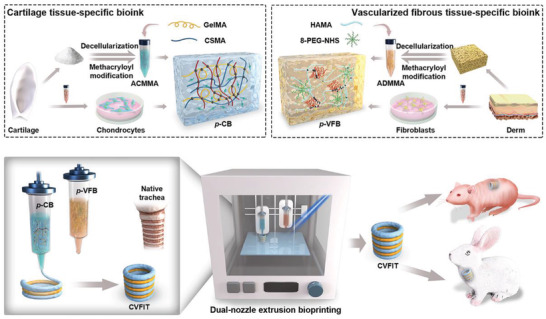
Schematic illustration of the designs of cartilage‐ and vascularized fibrous tissue‐specific bioinks and 3D‐bioprinted **CVFIT** for trachea regeneration in nude mice and in situ trachea reconstruction of rabbits. **CVFIT**: cartilage‐vascularized fibrous tissue‐integrated trachea; ***p*‐CB**: photocrosslinkable cartilage‐specific bioink; ***p*‐VFB**: photocrosslinkable vascularized fibrous tissue‐specific bioink.

Herein, we propose a novel strategy of 3D bioprinting to create a cartilage‐vascularized fibrous tissue‐integrated trachea (**CVFIT**), taking advantage of photocrosslinkable tissue‐specific bioinks meticulously designed for functional trachea reconstruction (**Figure** **1**). The alternant tubular design of **C** rings and **VF** rings could precisely mimic the physiological architecture and functions of the native trachea. The **C** rings were constructed by the chondrocyte‐loaded photocrosslinkable cartilage‐specific bioink (***p*‐CB**), consisting of methacryloyl‐modified gelatin, chondroitin sulfate, and cartilage acellular matrix (**GCC** hydrogel). The vascularized fibrous ring was constructed by the fibroblast‐loaded photocrosslinkable vascularized fibrous tissue‐specific bioink (***p*‐VFB**), consisting of methacrylate‐modified hyaluronic acid, 8‐arm‐polyethylene glycol‐succinic acid ester (**8‐PEG‐NHS**), and methacryloyl‐modified derm acellular matrix (**HPD** hydrogel). These specially designed multicomponent bioinks not only could meet the fundamental requirements of 3D bioprinting based on temperature‐sensitive (**GCC** hydrogel) and high‐viscosity (**HPD** hydrogel) properties, but also would mimic the tissue‐specific microenvironments of cartilage and vascularized fibrous tissue, respectively. The hybrid photoinitiated polymerization reaction occurring in both bioinks could offer rapid crosslinking and enhanced mechanical properties, while the amidation reaction mediated by **8‐PEG‐NHS** at the two‐phase interface was designed to strengthen the integration between the adjacent rings. Furthermore, the feasibility of trachea regeneration with proper mechanical and physiological functions was investigated by subcutaneous implantation of **CVFIT**. Finally, in situ trachea reconstruction was evaluated using the bioprinted and prematured trachea. Our innovative design provides a feasible strategy for functional trachea reconstruction with the alternant cartilage‐fibrous tissue‐mimicking architecture similar to that of the native trachea, implying a high feasibility of complex tissue regeneration based on 3D bioprinting for repair and reconstruction of complex tissue/organ defects in the future.

## 2 Results and Discussion

### 2.1 Characterizations of Photocrosslinkable Tissue‐Specific Bioinks

**Figure 2:**
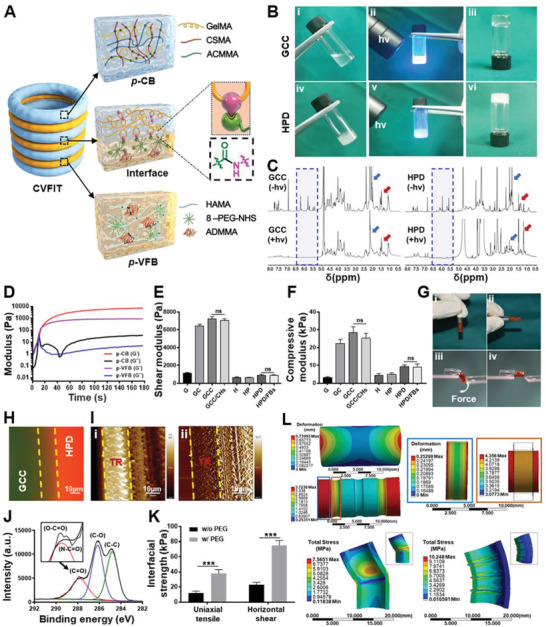
Characterizations of photocrosslinkable bioinks and interfacial bonding. A) Schematic of the alternant **CVFIT** structure constructed by photoinitiated polymerization reaction based on **GCC** and **HPD** hydrogels and amidation reaction for interfacial bonding. B) Photographs of sol‐to‐gel transition upon light irradiation (365 nm, 20 mW cm^−2^) within 10 s for i–iii) **GCC** and iv–vi) **HPD** formulations. C) ^1^H NMR spectra of photoinitiated polymerization reaction upon light irradiation of **GCC** and **HPD** formulations. Blue arrows represent proton peak of proteoglycan; red arrows represent proton peak of protein. D) Time‐sweep rheological analyses of **GCC** and **HPD** formulations showing the rapid gelation at approximately 5 s. E) Shear and F) compressive moduli of photocrosslinkable hydrogels with different compositions. (ns: no significance). G) Images of hydrogel interfacial force measurement experiments under i,ii) static and iii,iv) dynamic forces. Integrated hydrogels were not detached under force loading. **HPD** hydrogels were stained by rhodamine (red). Interfacial characterizations showing H) fluorescence staining and I) AFM (atomic force microscopy) images between **GCC** (fluorescein‐stained, green) and **HPD** (rhodamine‐stained, red) hydrogels. TR: **GCC**‐**HPD** transitional region. J) XPS (X‐ray photoelectron spectroscopy) analyses of the interfacial bonding with **8‐PEG‐NHS** treatment on **GelMA** gel surface. K) The interfacial strength of uniaxial tensile and horizontal shear of the interfacial bonding between **GCC** and **HPD** hydrogels. L) The finite element analyses of longitudinal tensile and lateral bending. The tested samples were obtained from the regenerated alternant structural trachea (RT) and the unitary cartilage tube (CT). **G**: 10% w/v GelMA gel; **GC**: 10% w/v GelMA/2% w/v CSMA gel; **GCC**: 10% w/v **GelMA**/2% w/v **CSMA**/1% w/v **ACMMA** gel; **H**: 2% w/v HAMA gel; **HP**: 2% w/v HAMA/5% w/v 8‐PEG‐NHS gel; **HPD**: 2% w/v **HAMA**/5% w/v **8‐PEG‐NHS**/1% w/v **ADMMA** gel. CHs: chondrocytes; FBs: fibroblasts.

Decellularized extracellular matrix (dECM) was chosen to mimic the microenvironments for preparation of tissue‐specific bioinks^[^ ^39^, ^40^, ^41^, ^42^, ^43^, ^44^ ^]^ and a photocrosslinking strategy was further utilized to allow stabilization of the tissue‐specific bioinks in facilitating 3D bioprinting.^[^ ^16^, ^17^, ^18^, ^19^, ^20^ ^]^ In this study, dECMs were obtained from porcine ear cartilage and dermal tissue to synthesize methacryloyl‐modified acellular cartilage matrix (**ACMMA**) and methacryloyl‐modified acellular derm matrix (**ADMMA**), respectively, where the successful graft of methacryloyl groups was confirmed by ^1^H nuclear magnetic resonance (NMR) (Figure S1A, Supporting Information). The glycosaminoglycan (GAG) and collagen content assessments before and after decellularization showed the partial loss of collagen and GAG in the **ACMMA** treated by collagenase digestion, while the partial loss of GAG in the **ADMMA** treated by pepsin digestion (Figure S2, Supporting Information). To better emulate the constitutions of cartilage and fibrous tissues, gelatin methacryloyl (**GelMA**)^[^ ^45^ ^]^ and methacrylate‐modified chondroitin sulfate (**CSMA**)^[^ ^46^ ^]^ were added into **ACMMA** to both supplement the partial loss of collagen and GAG during decellularization as well as to enable formulation of the photocrosslinkable cartilage‐specific bioink (i.e., **GCC**) (**Figure** **2**A). Meanwhile, the combination of methacrylate‐modified hyaluronic acid (**HAMA**),^[^ ^47^ ^]^ **ADMMA**, and **8‐PEG‐NHS** enabled formulation of the photocrosslinkable vascularized fibrous tissue‐specific bioink (i.e., **HPD**). **HAMA** was added to supplement the partial loss of GAG during decellularization; while **8‐PEG‐NHS** was further incorporated to react with amino groups of **GelMA** in the **GCC** hydrogel by the amidation reaction^[^ ^48^, ^49^ ^]^ for ensuring interfacial bonding between **GCC** and **HPD** gels. Collectively, the proteoglycan and collagen contents in the **GCC** gels and **HPD** gels were recovered to the levels close to those of the native trachea cartilage and connective tissue, respectively (Figure S2, Supporting Information).

Next, rheological analyses were conducted to investigate the gelling properties by in situ photorheometry. As shown in Figure 2B‐D, and Video S1 (Supporting Information), time‐dependent sweep tests exhibited the fast gelation at approximately 8 s for both **GCC** and **HPD** bioinks, implying the quick crosslinking of photoinitiated polymerization reaction. Additionally, the photocuring thickness of both **GCC** and **HPD** bioinks could reach more than 2 cm depth upon 365 nm LED irradiation for 30 s with light intensities beyond 20 mW cm^−2^ (Figure S3, Supporting Information). Then, ^1^H NMR spectra were used to analyze the crosslinking reaction, and the signals of gel precursor distinctly decreased at 5.1–6.3 ppm, which confirmed the successful polymerization among methacryloyl groups of the multicomponent polymers (Figure 2C). The corresponding shear moduli of the hydrogels made with different formulations were further compared. As shown in Figure 2E, the addition of **CSMA** and **ACMMA** into the **GelMA** gels obviously increased the shear modulus from 1122±98 Pa (**G**: 10% w/v **GelMA** gel) to 6453±176 Pa (**GC**: 10% w/v **GelMA**/2% w/v **CSMA** gel) and 7215±273 Pa (**GCC**: 10% w/v **GelMA**/2% w/v **CSMA**/1% w/v **ACMMA** gel); the addition of **8‐PEG‐NHS** into the **HAMA** gels had no significant difference in shear modulus (**H**: 2% w/v **HAMA** gel, 654±98 Pa; **HP**: 2% w/v **HAMA**/5% w/v **8‐PEG‐NHS** gel, 623±65 Pa), while the addition of **ADMMA** slightly enhanced the shear modulus to 886±76 Pa (**HPD**: 2% w/v **HAMA**/5% w/v **8‐PEG‐NHS**/1% w/v **ADMMA** gel). Consistent with the rheological tests, the compressive moduli of **GCC** and **HPD** gels also displayed a significantly increasing tendency compared to single‐component gels (**G** and **H** gels) due to the enhanced multicrosslinked networks^[^ ^50^ ^]^ (Figure 2F). Notably, the encapsulation of cells into **GCC** or **HPD** hydrogels had no significant effect on shear or compressive modulus. In addition, the hydrogels possessed relatively small swelling ratios (**GCC**: 113.4±4.6%; **HPD**: 103.6±5.2%), presented porous structures after freeze‐drying, and could be mostly degraded in the mixed enzyme solution (20 U mL^−1^ of hyaluronidase and 20 U mL^−1^ of collagenase) within 7 d (Figure S4, Supporting Information).

### 2.2 Analyses of Interfacial Bonding and Structural Design

Furthermore, the interfacial bonding between **GCC** and **HPD** gels, which was essential to maintaining integrity of the alternant ring‐to‐ring tubular structure, was analyzed qualitatively and quantitatively. As shown in Figure 2G and Video S1 (Supporting Information), these two hydrogels tightly interfaced with each other and still retained reliable integration under lateral force. The microscopic images of fluorescent staining and atomic force microscopy (AFM) further confirmed the seamless interfacial fusion, of which the macromolecules in the **GCC** gel permeated into the **HPD** gel forming a stiff‐to‐soft transitional layer (Figure 2H,I). The X‐ray photoelectron spectroscopy (XPS) results revealed that the **GelMA** hydrogel samples treated with the **8‐PEG‐NHS** adhesive had a larger peak area of C‐O (286.2 eV) species than bare **GelMA**, consistent with the fact that **PEG** contained more glycol groups (Figure S1B, Supporting Information). Additionally, a new component associated with amide bond appeared at 287.6 eV, indicating that the enhanced interfacial strength attributed to the amidation reaction (Figure 2J). Quantitative analyses of uniaxial tensile and horizontal shear strength between **GCC** and **HPD** gels further confirmed that the interfacial adhesion strength in **PEG**‐treated groups reached over threefold compared to those of nontreated groups (Figure 2K). The effect of alternant stiff‐to‐soft structure (the tested samples from the regenerated alternant structural trachea and the unitary cartilage tube; shown in Figure 5A) on deformation and stress distributions were also evaluated by finite element analysis. As shown in Figure 2L, the alternant tubular stiff‐to‐soft structure exhibited longitudinal stretchability and lateral flexibility, whereas the unitary cartilage tube was difficult to longitudinally stretch and easily caused stress concentration in the central region during lateral bending. All these results demonstrated that the alternant stiff‐to‐soft architecture with interfacial chemical bonding facilitated to realize physiological mechanical functions of the regenerated trachea.

### 2.3 Biological Evaluation of Photocrosslinkable Tissue‐specific Bioinks

**Figure 3:**
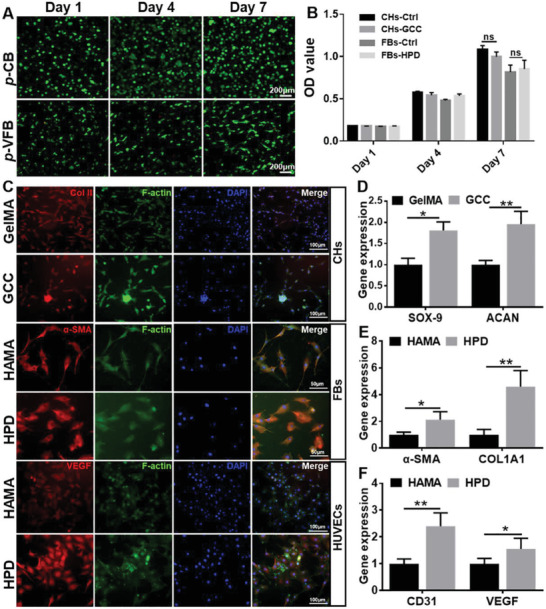
Biological evaluation of photocrosslinkable tissue‐specific bioinks. A) Live/dead staining and B) CCK‐8 tests of chondrocyte‐loaded **GCC** and fibroblast‐loaded **HPD** hydrogels incubated for 1, 4, and 7 d. C) Immunofluorescent staining of COL II (red), F‐actin (green), and cell nuclei (DAPI, blue) for chondrogenic marker expressions in **G** and **GCC** groups; *α*‐SMA (red), F‐actin (green), and cell nuclei (DAPI, blue) for fibrogenic marker expressions in **H** and **HPD** groups; VEGF (red), F‐actin (green), and cell nuclei (DAPI, blue) for vascularization marker expressions in **H** and **HPD** groups. D–F) Comparative chondrogenic gene expressions (*ACAN*, *SOX 9*) for cartilage‐specific hydrogels, as well as fibrogenic gene expressions (*COL I*, *α‐SMA*) and angiogenic gene expressions (*VEGF*, *CD31*) for vascularized fibrous tissue‐specific hydrogels (*n* = 4, *p < 0.05, **p < 0.01). HUVECs: human umbilical vein endothelical cells.

Cytocompatibility is the essential evaluation for bioinks. As shown in **Figure** **3**A,B, cell counting kit‐8 (CCK‐8) and live/dead staining demonstrated that neither **GCC** nor **HPD** gel showed noticeable cytotoxicity (cell viability >95%), indicating satisfactory cytocompatibility of the biomimetic hydrogels. In addition, the tissue‐specific fluorescence staining results revealed that cartilage‐related expression of type II collagen (Col II) in chondrocytes were stronger in the **GCC** gel than that in the **GelMA** gel (Figure 3C). The fibrous tissue‐related expression of *α*‐smooth muscle actin (*α*‐SMA) in fibroblasts and blood vessel‐related expression of vascular endothelial growth factor (VEGF) in endothelial cells were stronger in the **HPD** gel than those corresponding expressions in the **HAMA** gel (Figure 3C). The real‐time quantitative polymerase chain reaction (*q*‐PCR) results further confirmed that the chondrogenic gene expressions (*ACAN*, *SOX 9*) in ***p*‐CB**, as well as fibrogenic gene expressions (*COL I*, *α‐SMA*) and angiogenic gene expressions (*VEGF*, *CD31*) in ***p*‐VFB**, were significantly higher than those in the corresponding control groups (Figure 3D). All these results demonstrated that the biomimetic hydrogels had definitive tissue‐specific regulatory functions due to their successful mimicking of chondrogenic and fibrogenic/angiogenic microenvironments.

### 2.4 Printability Evaluation of Bioinks and Characterizations of Bioprinted CVFIT

**Figure 4:**
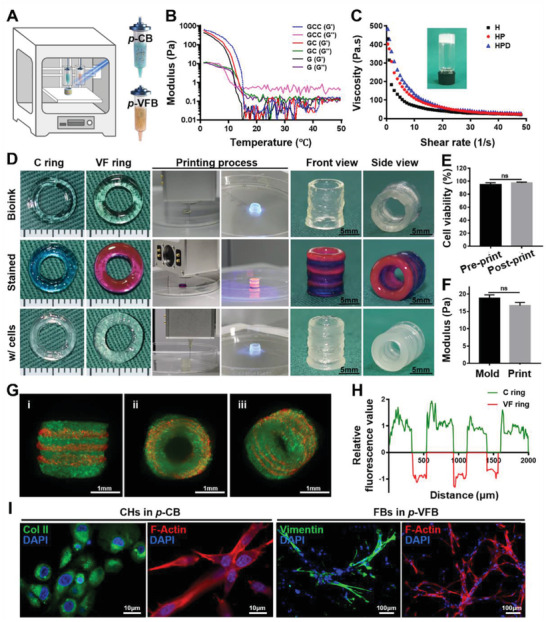
Printability evaluation of bioinks and characterizations of bioprinted **CVFIT**. A) Schematic of the dual‐nozzle 3D extrusion‐based bioprinting for **CVFIT** construction based on tissue‐specific bioinks of ***p*‐CB** and ***p*‐VFB**. B) Temperature‐sweep rheological analyses indicating the printability of ***p*‐CB** based on its temperature‐sensitive property. C) Viscosity profiles implying the printability of ***p*‐VFB** based on its relatively high viscosity and shear‐thinning properties. D) Photographs of the 3D‐printed trachea‐analogues, dye‐stained bioinks for visualization, and bioprinted **CVFIT** using cell‐loaded bioinks. E) Cell viability and F) mechanical properties of bioinks after molding or 3D printing. G) Lightsheet microscopy images for visualizing cell distributions in the bioprinted **CVFIT**. GFP‐labeled chondrocytes (green); RFP‐labeled fibroblasts (red). H) Relative fluorescence intensity analyses of the bioprinted **CVFIT** using ImageJ. I) Immunofluorescent staining of Col II for chondrocytes in ***p*‐CB**, and vimentin for fibroblasts in ***p*‐VFB**. **CVFIT**: cartilage‐vascularized fibrous tissue‐integrated trachea; ***p*‐CB**: photocrosslinkable cartilage‐specific bioink; ***p*‐VFB**: photocrosslinkable vascularized fibrous tissue‐specific bioink;**C** ring: cartilage ring; **VF** ring: vascularized fibrous tissue ring.

The printability of bioinks is the most important requirement for constructing complex structures through 3D extrusion bioprinting (**Figure** **4**A). Temperature‐dependent sweep and viscosity evaluations were conducted to assess the printability of the bioinks. As shown in Figure 4B, the **G**, **GC**, and **GCC** formulations showed temperature‐dependent gel‐transition points around 12–16 °C, implying that the hydrogels containing the temperature‐sensitive **GelMA** component could be used for bioprinting under appropriately 16 °C.^[^ ^51^ ^]^ Meanwhile, the **H**, **HP**, and **HPD** formulations exhibited high viscosities and shear‐thinning nature due to the addition of the high‐molecular weight **HAMA** (1400 kDa) component, and thus facilitated extrusion of the gel precursor without any collapse during bioprinting (Figure 4C). The performance of printed lines was further tested to obtain optimal printing parameters, such as printing speed, pressure, and nozzle size (Figure S5, Supporting Information). Collectively, the inherent thermosensitivity of the **GCC** formulation and the suitable viscosity of the **HPD** formulation were verified as two feasible strategies for extrusion‐based 3D bioprinting.

Then, using ***p*‐CB** and ***p*‐VFB** bioinks along with a dual‐nozzle 3D extrusion bioprinting system, trachea analogue featuring the alternating ring‐to‐ring tubular structure was successfully bioprinted, followed by light irradiation (365 nm, 20 mW cm^−2^) within 30 s for further photocrosslinking to enhance the mechanical strength and maintain the designed shape (Figure 4D). The stained trachea analogue distinctly displayed the alternating ring‐to‐ring tubular structure with satisfactory interfacial integration (**C** rings: blue; **VF** rings: red). Specifically, the alternant **C** ring‐**VF** ring tubular structure containing localized chondrocytes and fibroblasts was successfully bioprinted based on ***p*‐CB** and ***p*‐VFB**, and the bioprinted **CVFIT** maintained a stable tubular shape with satisfactory mechanical strength and elasticity (Figure 4D and Video S2, Supporting Information). In addition, the shearing force during the extruding process had no obvious influence on cell viability (Figure 4E) and the mechanical properties also did not show significant differences with or without 3D bioprinting (Figure 4F). In the 3D‐bioprinted trachea‐analogue, the fluorescently labeled chondrocytes (green) and fibroblasts (red) alternately distributed in the ring‐to‐ring structure without intruding each other, confirming the precise control of 3D bioprinting on both units and cell localization (Figure 4G,H and Video S3, Supporting Information). Importantly, the fluorescence staining results showed the spreading cell morphology (F‐actin) and tissue‐specific protein expressions for chondrocytes (Col II) in the **GCC** gel and fibroblasts (vimentin) in the **HPD** gel after 14 d of culture in vitro, again indicating the satisfactory phenotype‐maintenance in these tissue‐specific hydrogels (Figure 4I). Additionally, the complex human‐sized 3D‐printed tissue‐mimics, such as auricle, meniscus, and trachea, could also be successfully printed, which exhibited similar overall geometries to native tissues (Figure S6, Supporting Information). All these results demonstrated that the dual‐nozzle 3D extrusion bioprinting technique in conjunction with our specially designed photocrosslinkable bioinks could be used to accurately construct cartilage‐fibrous tissue‐alternating biomimetic trachea in a cell‐friendly manner.

### 2.5 In Vivo Subcutaneous Implantation of Bioprinted CVFIT in Nude Mice

**Figure 5:**
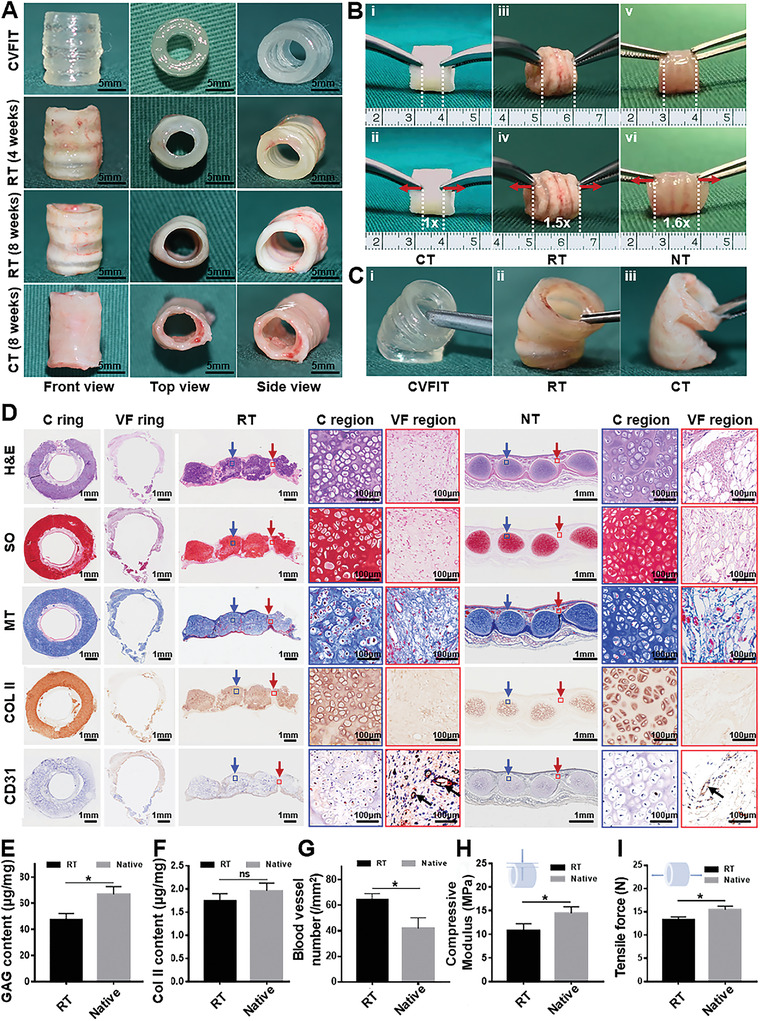
In vivo subcutaneous implantation of bioprinted **CVFIT** in nude mice. A) Gross view of the bioprinted **CVFIT**, regenerated trachea, and unitary cartilage tube after 4 or 8 weeks of implantation. B) Mechanical function evaluations of longitudinal tensile and C) lateral bending tests. CT: cartilage tube; RT: regenerated trachea; NT: native trachea. D) Histological examinations of H&E, safranin‐O (SO), Masson's trichrome (MT), type II collagen (COL II), and CD31 staining of the regenerated trachea in the transverse and longitudinal sections at 8 weeks and of the native counterpart. Blue arrows represent cartilage regions; red arrows represent vascularized fibrous tissue regions; black arrows represent blood vessels. Quantitative analyses of the E) GAG contents, F) COL II contents, G) blood vessel numbers, H) Compressive moduli, and I) tensile forces of the regenerated and native trachea (*n* = 4, *p < 0.05, ns: no significance).

To further investigate the feasibility of trachea regeneration, the **CVFIT** prints (15 mm in height; 8 mm in external diameter; 6 mm in inner diameter; four chondrocyte‐loaded **C** rings and three fibroblast‐loaded **VF** rings; height ratio **C**:**VF** = 2:1) were subcutaneously implanted in nude mice with an isometric silicone tube as the internal mechanical support, while the chondrocyte‐loaded tubular prints (no connective tissue rings) were used as the control. After 8 weeks, the tubular shape and the cartilage‐fibrous tissue‐alternating structure were still largely maintained similar to that of the native trachea, whereas the cartilage‐only tube in the control group exhibited the single cartilage structure (**Figure** **5**A). Noticeably, due to the alternant stiff‐to‐soft tissue structure, the regenerated trachea showed satisfactory compressive resistance, longitudinal stretchability, and lateral flexibility close to those of the native trachea, which obviously facilitated to reconstruct normal mechanical functions of the trachea (Figure 5B and Video S4, Supporting Information). Consistent with the results of finite element analysis (Figure 2L), due to the single stiff cartilage tissue structure, the regenerated cartilage‐only tube lacked longitudinal stretchability and presented visible structural deficiency under lateral stress loading, which inevitably had a negative effect on smooth ventilation during dynamic movements, such as raising and bowing the head as well as laterally bending the neck, as simulated and shown in Video S5 (Supporting Information).

The histological examinations of the transverse and longitudinal sections further confirmed that the regenerated biomimetic trachea showed clearly alternant cartilage‐fibrous tissue structure similar to that of the native trachea (Figure 5C). More importantly, tissue‐specific regeneration with seamless interfacial integration was observed, which presented a gradual maturation tendency from 2 weeks to 8 weeks along with the degradation of the hydrogels (Figure S7, Supporting Information). After 8 weeks, the cartilage region exhibited the typical lacuna structure and cartilage‐specific ECM deposition (safranin‐O and COL II), while the fibrous tissue region presented the typical connective tissue features with visible blood vessel infiltration and negative cartilage‐specific staining. The CD31 staining further confirmed that angiogenesis occurred only in the fibrous tissue region but not in the cartilage region (Figure 5D). Differently, the regenerated unitary cartilage tube exhibited relatively inferior cartilage regeneration (especially in the inner wall) in terms of cartilage thickness and structural integrity also with negative blood vessel staining, which should be attributed to the lack of enough nutrition supply due to the design deficiency in vascular fibrous rings and inherent avascular feature of the cartilage. In other words, the alternant cartilage‐vascularized fibrous tissue structural design ensured that the cartilage rings could obtain sufficient nutrition supply from the adjacent vascularized fibrous rings, which was an important reason for superior cartilage regeneration in our experimental trachea compared to the control, unitary cartilage tube (Figures S8 and S9, Supporting Information). Quantitative analyses confirmed that the biochemical ECM contents and mechanical properties (compression and tensile evaluations) of the regenerated biomimetic trachea were close to those of the native trachea (Figures 5E–I and S10, Supporting Information).

Collectively, the bioprinted trachea with alternant stiff‐to‐soft tissue structure not only facilitated mechanical functional recovery (compressive resistance, tensile, and bending ability), but also benefited the physiological functional reconstruction due to the sufficient nutrition supply provided by the vascularized fibrous tissue rings.

### 2.6 Segmental Trachea Reconstruction Surgery and Therapeutic Outcome

**Figure 6:**
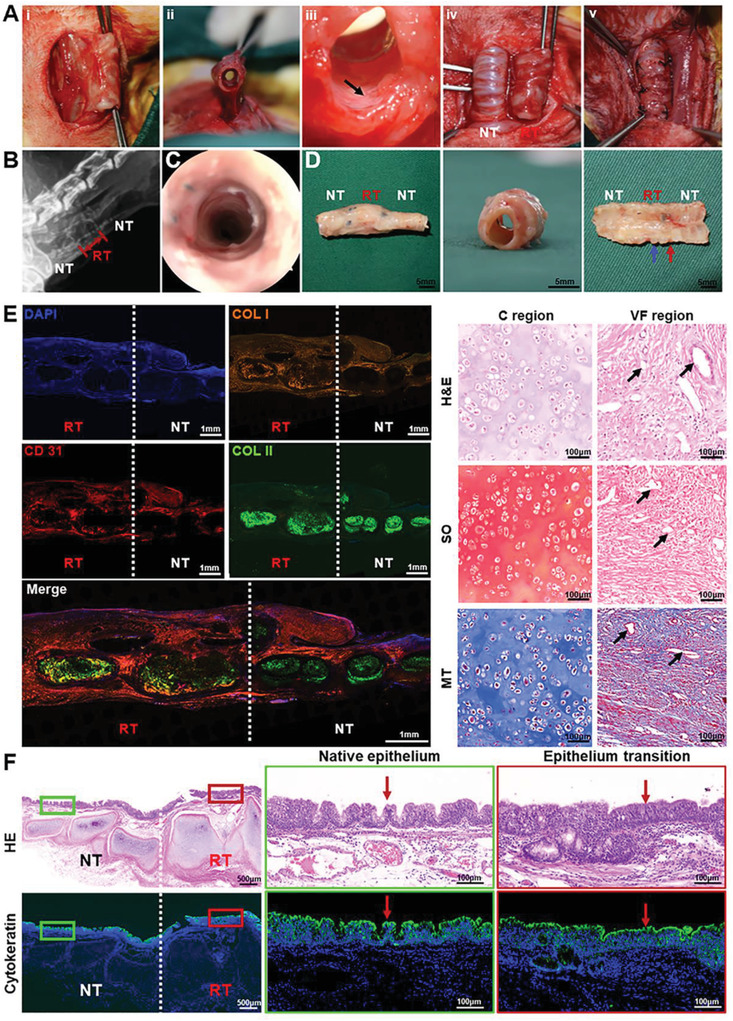
Segmental trachea reconstruction surgery and therapeutic outcome. A‐i,ii) Photographs of regenerated trachea with the muscle pedicles, iii) well‐vascularized inner wall of regenerated trachea, iv) exposed regenerated and native trachea, and v) end‐to‐end anastomosis of regenerated trachea with cut ends of the native trachea. Black arrows represent blood vessels. B) X‐ray and C) tracheoscopy images of the in situ repaired trachea segment at 8 weeks postsurgery. D) Gross view of the reconstructed trachea segment at 8 weeks postsurgery. Blue arrows represent the regenerated cartilage ring; red arrows represent regenerated vascularized fibrous ring. E) Histological staining of H&E, Safranin‐O (SO), Masson's trichrome (MT), and immunofluorescence staining of type II collagen (COL II, green), type I collagen (COL I, orange), CD31 (red), and cell nuclei (DAPI, blue). Black arrows represent blood vessels. F) H&E staining and immunofluorescence staining of cytokeratin (green) and cell nuclei (DAPI, blue) showing the regeneration of tracheal epithelium. Red arrows represent mucosaepithelium. RT: regenerated trachea; NT: native trachea.

To finally verify the feasibility of functional reconstruction using our bioprinted biomimetic trachea, the segmental tracheal defect (15‐mm‐long) repair was conducted in a rabbit model.^[^ ^11^, ^12^ ^]^ The bioprinted **CVFIT** was preimplanted and wrapped by a vascular muscle flap in the rabbit neck, followed by the repair of the tracheal defect using the regenerated trachea (8 week preculture in vivo) along with the vascular muscle pedicle, via end‐to‐end anastomosis (**Figure** **6**A). As shown in Figure 6B,C, and Video S6 (Supporting Information), X‐ray and tracheoscopy examinations showed the airway patency and functional recovery (smooth ventilation) of the repaired trachea. At 8 weeks postsurgery, gross view suggested that the reconstructed trachea presented a complete and continuous tubular structure with a smooth inner surface, had no sputum accumulation, and showed satisfactory integration with the native trachea (Figure 6D). Histological examinations further revealed an alternant cartilage‐fibrous tissue structure: mature cartilage tissue with the typical lacunar structure and abundant cartilage‐specific ECM deposition in the cartilage region; typical vascularized fibrous tissue with collagen deposition and plentiful blood vessel infiltration in the vascularized fibrous region (Figure 6E). In addition, the regenerated cartilage rings exhibited seamless integration with the surrounding vascularized fibrous rings, and the reconstructed trachea also exhibited good connection with the native trachea, which was obviously essential to the functional recovery required for smooth ventilation (Figure S11, Supporting Information). More importantly, the epithelium‐like tissue with positive staining of cytokeratin (specific phenotype of trachea epithelium) was distinctly observed on the luminal surface of the reconstructed trachea, indicating successful epithelium regeneration, which apparently helped to recovery of tracheal physiological functions (Figure 6F). All these results demonstrated that our rationally designed, bioprinted, biomimetic trachea could achieve desired functional reconstruction with alternant cartilage‐vascularized fibrous tissue‐regeneration as well as epithelialization similar to the native trachea.

## 3 Conclusions

In summary, the current study developed a novel strategy for functional trachea reconstruction using a 3D‐bioprinted biomimetic cartilage‐vascularized fibrous tissue‐integrated trachea. The photocrosslinkable tissue‐specific bioinks meticulously designed by us provided suitable printability, satisfactory biocompability, and biomimetic microenvironments for chondrogenesis and vascularized fibrogenesis based on the multicomponent synergistic effects. More importantly, the tubular analogues featuring alternant cartilage and vascularized fibrous tissue rings were successfully bioprinted and the ring‐to‐ring architecture was tightly integrated through chemically enhanced interfacial bonding. The functional trachea reconstruction in both mechanical and physiological characteristics was successfully achieved due to the alternant stiff‐to‐soft tissue structure, which were very close to that of the native trachea. This study thus suggests the 3D‐bioprinted native tissue‐like trachea as a promising alternative for future clinical trachea reconstruction.

## 4 Experimental Section

### Materials and Animals

In this study, gelatin, chondroitin sulfate, hyaluronic acid, lithium phenyl‐2,4,6‐trimethylbenzoylphosphinate (LAP), trypsin, collagenase, pepsin, Triton X‐100, methacrylic anhydride, and sodium hydroxide were purchased from Sigma‐Aldrich. 8‐PEG‐NHS (10 kDa) was purchased from JenKem Company. All the other chemicals were reagent grade and deionized water was used. Both nude mice and New Zealand white rabbits were purchased from Shanghai Jiagan Experimental Animal Raising Farm. All protocols for experimental in animals were approved by the Animal Care and Experimental Committee of Shanghai Jiao Tong University School of Medicine (SH9H‐2021‐A655‐SB).

### ACMMA and ADMMA Syntheses

Total cartilage derived from porcine ear and skin tissues were obtained from local butchers to prepare **ACMMA** and **ADMMA**. The photocrosslinkable dECMs were synthesized according to the following three steps:

i) Powder‐like dECM preparation:^[^ ^52^ ^]^ the fresh tissue was first grounded into powder by freezer mixer, and treated with 0.5% trypsin/phosphate‐buffered saline (PBS) (w/v) for 24 h. The samples were then sequentially treated with nuclease solution (containing 50 U mL^−1^ of deoxyribonuclease and 1 U mL^−1^ of ribonuclease A in 10 × 10^−3^ m of Tris‐HCL, pH = 7.5) for 4 h, 10 × 10^−3^ m Tris‐HCL (including 10 U mL^−1^ of aprotinin) for 20 h, and 1% Triton X‐100/PBS solution (v/v) for 24 h. Then, the powder‐like dECM was prepared by freeze‐drying after washing 3 times in deionized water.
ii) Water‐soluble treatment of dECM: cartilage‐derived dECM was treated with 0.15% collagenase, and skin‐derived dECM were treated with 0.2% trypsin (0.1‐m HCl solution) for 24 h, respectively. Then, the solution was dialyzed against deionized water for 3 d and followed by freezing and lyophilizing to obtain water‐soluble dECM.
iii) Methacryloyl‐modification of dECM: 0.5 g water‐soluble dECM was dissolved in deionized water and 0.5 mL of methacrylic anhydride was added dropwise into the above solution in an ice bath. The pH was maintained between 8 and 11 adjusted by 5 m NaOH (aq) and the reaction continued overnight in the dark at 0–4 °C. After the reaction, the solution was centrifuged by centrifugation (5000 rpm) to remove insoluble substances. The pH of the solution was adjusted to 7.4 by 1‐m HCl (aq). Then, the crude product was dialyzed against deionized water for 3 d followed by freezing and lyophilizing. ^1^H NMR analysis was performed to determine the substitution degree of methacryloyl modification.

### GelMA Synthesis

10 g of gelatin was dissolved in 500 mL PBS (pH = 7.4) and stirred vigorously at 50 °C until complete dissolution. 10 mL methacrylate anhydride was slowly added into the above solution and reacted with gelatin for 2 h. After the reaction, the solution was collected and insoluble substances were removed by centrifugation (5000 rpm). Then, the crude product was dialyzed against deionized water at 40 °C for 3 d followed by freezing and lyophilizing. ^1^H NMR analysis was performed to determine the substitution degree of methacryloyl modification as previously described.^[^ ^45^ ^]^

### CSMA Synthesis

10 g of chondroitin sulfate (500 kDa) was dissolved in 500 mL of deionized water and stirred vigorously until complete dissolution. 10 mL of methacrylic anhydride was added dropwise to the above solution in an ice bath. The pH was maintained between 8 and 11 adjusted by 5‐m NaOH (aq) and the reaction continued overnight in the dark at 0–4 °C. After the reaction, the solution was centrifuged by centrifugation (5000 rpm) to remove insoluble substances. The pH of the solution was adjusted to 7.4 by 1‐m HCl (aq). Then, the crude product was dialyzed against deionized water for 3 d followed by freezing and lyophilizing. ^1^H NMR analysis was performed to determine the substitution degree of methacrylation as previously described.^[^ ^46^ ^]^

### HAMA Synthesis

5 g of hyaluronic acid (1400 kDa) was dissolved in 500 mL of deionized water and stirred vigorously until complete dissolution. 20 mL methacrylic anhydride was added dropwise to the above solution in an ice bath. The pH was maintained between 8 and 11 adjusted by 5‐m NaOH (aq) and the reaction continued overnight in the dark at 0–4 °C. After the reaction, the solution was centrifuged by centrifugation (5000 rpm) to remove insoluble substance. The pH of the solution was adjusted to 7.4 by 1‐m HCl (aq). Then, the crude product was dialyzed against deionized water for 3 d followed by freezing and lyophilizing. ^1^H NMR analysis was performed to determine the substitution degree of methacrylation as previously described.^[^ ^47^ ^]^

### Hydrogel Preparation

Hydrogel precursors of **GelMA**, **CSMA**, **ACMMA**, **HAMA**, **8‐PEG‐NHS**, **ADMMA** and LAP (0.2% w/v) were mixed according to different requirements in Dulbecco's PBS (D‐PBS, pH 7.4). Then, the above samples were subjected to different measurements following light irradiation (365‐nm LED, 20 mW cm^−2^) for 1 min. The hydrogel composition in this study were listed as follows: **G** gel: 10% w/v **GelMA**; **GC** gel: 10% w/v **GelMA**/2% w/v **CSMA**; **GCC** gel: 10% w/v **GelMA**/2% w/v **CSMA**/1% w/v **ACMMA**; **H** gel: 2% w/v **HAMA**; **HP** gel: 2% w/v **HAMA**/5% w/v **8‐PEG‐NHS**; **HPD** gel: 2% w/v **HAMA**/5% w/v **8‐PEG‐NHS**/1% w/v **ADMMA**. (w/v: weight/volume).

To clearly observe their morphologies, the dehydrated hydrogel samples were coated with gold‐palladium in a Hitachi S‐3400N ion sputter. The morphologies of hydrogels with different components were analyzed using scanning electron microscopy (SEM, Philips XL‐30) at an accelerating voltage of 10 kV.

### Component Analyses of Hydrogel Precursors

The GAG content was estimated via quantifying the amount of sulfated glycosaminoglycans using the 1,9‐dimethylmethylene blue reagent (DMMB, Sigma‐Aldrich). The absorbance was measured with microplate reader (Synergy H1, BioTek) at the wavelength of 492 nm. The standard curve was produced using chondroitin sulfate A as standard samples in advance. The total collagen content was determined via a universal hydroxyproline assay kit (Sigma‐Aldrich). The absorbance of the samples was measured at wavelength of 550 nm and quantified by referring to a standard curve produced using hydroxyproline in advance.

### Rheological Analyses of Hydrogel Precursors

Dynamic rheology experiments were performed on the HAAKE MARS III photorheometer with parallel‐plate (P20 TiL, 20 mm diameter) geometry and OmniCure Series 2000 (365 nm, 20 mW cm^−2^) at 25 °C. Time‐sweep oscillatory tests were performed at a 10% strain (CD mode), 1 Hz frequency, and a 0.5 mm gap for 180 s. Strain sweep oscillatory tests were performed to verify the linear response. The gel point was determined as the time when the storage modulus (G’) surpassed the loss modulus (G’’). The final shear modulus was determined as the storage modulus (G’) reaching to the complete gelation. Temperature‐sweep oscillatory tests were performed at 10% strain (CD mode) and 1 Hz frequency parameters from 50 to 0 °C. The sol‐to‐gel transition point was determined as the time when the storage modulus (G’) surpassed the loss modulus (G’’). Viscosity tests were performed at a gradual increasing shear rate from 0 to 50 s^−1^.

### Mechanical Measurements of Hydrogels

Mechanical tests were carried out on as‐prepared hydrogel samples (cylindrical shape with 10 mm diameter and 3 mm height) using the GT‐TCS‐2000 universal material testing machine with a capacity of 100 N. For compression tests, the hydrogel samples were set on the test platform, and the testing speed was set at 1 mm min^−1^. The compressive modulus was calculated as the slope of the linear region (20–40% strain). The hydrogels were subjected to compression tests after complete gelation upon light irradiation (365 nm LED, 20 mW cm^−2^) for 1 min.

### Swelling and Degradation Tests

The cylindrical hydrogel samples (diameter = 10 mm; height = 2 mm) were recorded for the initial weights (W_0_). For swelling tests, the hydrogels were fully immersed in PBS solution (pH = 7.4) for 24 h until complete swelling (*n* = 4). When the masses of these hydrogels became constant, the values were recorded as the wet weights (*W*_t_). The swelling ratio was calculated according to Equation (1)

(1) $$\mathrm{Swelling}\;\;\mathrm{Ratio}\;\;\left(\%\right)=W_{t}/W_{0}\times 100\%$$

For enzyme‐mediated degradation tests, the above hydrogels after complete swelling were recorded for the initial weights (*W*_0_). Then, the hydrogels were incubated in PBS (pH = 7.4) supplemented with 20 U mL^−1^ of hyaluronidase and 20 U mL^−1^ of collagenase for observing enzyme‐mediated degradation at 37 °C (*n* = 4). The culture solution was refreshed every day to maintain the enzyme activity. At each time point, these samples were carefully collected, and gently blotted with filter paper to remove excess water on the surfaces, and recorded for the residual weights (*W*_t_). The degradation ratio (%) was calculated according to Equation (2)

(2) $$\mathrm{Degradation}\;\;\mathrm{ratio}\;\;\left(\%\right)=W_{t}/W_{0}\times 100\%$$

### Characterizations of Interfacial Bonding

To qualitatively analyze the interfacial bonding of the **GCC‐HPD** hydrogels, fluorescence microscopy, AFM, and XPS were conducted.

The **GCC‐HPD** hydrogel samples were prepared using a polytetrafluoroethylene mold with a cylindrical shape of 10 mm diameter and 4 mm height (2 mm height for each layer). The dual‐layered hydrogel samples were cut from intermediate region for the following characterization. For the visualization of interfacial region, the **GCC** hydrogel was stained with fluorescein (green), and the **HPD** hydrogel was stained with rhodamine (red). Confocal laser scanning microscopy (CLSM; Leica TCS SP8 STED 3X) was used to directly observe the interfacial condition. For atomic force microscope observation, the **GCC‐HPD** hydrogel samples were dried at 40 °C for 12 h, and followed by morphology analysis using Bruker MFP‐3D AFM.

The **GelMA** hydrogel samples were first formed upon light irradiation (365 nm LED, 20 mW cm^−2^) for 1 min. Then, 0.1% w/v **8‐PEG‐NHS** adhesive solution was applied to soak the **GelMA** hydrogel samples or without treatment as control. Then, the samples were dried at 40 °C for 12 h. Film characterization using XPS was carried out in an ultrahigh vacuum chamber by an ESCALAB 250Xi XPS system. Then, XPS spectra were analyzed by the XPSPEAK software to conduct peak separation.

### Measurements of Interfacial Strength

The hydrogel samples were prepared in a polytetrafluoroethylene mold with a cylindrical shape of 10 mm in diameter and 4 mm in height. The two types of the hydrogel precursors were added into the above mold in sequence, and followed by light irradiation (365‐nm LED, 20 mW cm^−2^) for 1 min to obtain a complete dual‐layered hydrogel samples (2 mm in height for each layer). Then, the hydrogel samples were glued to plastic sheets for tensile and shear tests. One side of the plastic sheet was fixed, and increasing tensile or shear force was applied. When the interface was separated, the mass of the tensile or shear force was recorded (*n* = 4). The interfacial strength was calculated according to Equation (3)

(3) $$\mathrm{Interfacial}\;\;\mathrm{strength}=F_{\max}/A$$

where *F*_max_ is the maximal tensile or shear stress and *A* is the cross‐sectional area.

### FEM Analyses of Unitary and Alternant Tubular Structure

The samples were obtained from the regenerated alternant structural trachea and the unitary cartilage tube (shown in Figure 5A). The size of regenerated trachea was listed as follows: 15 mm in height; 8 mm in external diameter; 6 mm in inner diameter; four **C** rings and three **VF** rings; height ratio **C**:**VF** = 2:1. The size of cartilage tube was listed as follows: 15 mm in height; 8 mm in external diameter; 6 mm in inner diameter. The mechanical behavior of the regenerated cartilage tube and alternant trachea under longitudinal tensile and lateral bending were analyzed using an FEM program (Ansys software). The alternant structural trachea was divided into stiff units (**C** rings) and soft units (**VF** rings), while the cartilage tube was the whole stiff unit. The Young's modulus and Poisson's ratio used for FEM analyses were obtained from the mechanical tests of the corresponding units in advance. The same stress in longitudinal tensile (forces on two ends) was adopted to compare the deformation distribution of different structural trachea. The same displacement in lateral bending test was applied on one end (another end was fixed) to achieve a 45° bending angle for comparing the stress distribution. To simplify the FEM analyses, a linear elastic model was adopted for approximately evaluating the structural and functional differences in this study.

### Cell Viability and Proliferation

Rabbit auricles were obtained from autologous rabbit under aseptic conditions. Chondrocytes were isolated from the rabbit auricular cartilage tissue. Fibroblasts were isolated from auricular skin dermis. The cells were expanded to the second passage according to a previously established method.^[^ ^26^ ^]^ The hydrogels were sterilized by filtration through a 0.22‐µm filter. Chondrocytes and fibroblasts were evenly mixed with hydrogels at a density of 1.0 × 10^7^ cells mL^−1^ at 37 °C. After 1, 4, and 7 d of culture, the cell viability of cell‐laden hydrogel constructs was evaluated using the Live & Dead Cell Viability Assay (Invitrogen) following the manufacturer's instructions and examined using a CLSM (Leica TCS SP8 STED 3X). Cell proliferation was examined using CCK‐8 (Dojindo) according to the manufacturer's protocol, and the optical density (OD) was measured with microplate reader (Synergy H1, BioTek) at wavelength of 450 nm.

### Chondrogenic, Fibrogenic, and Angiogenic Evaluations

Chondrocytes were incubated with **GelMA** and **GCC** hydrogels for 7 d, while fibroblasts and human umbilical vein endothelial cells (HUVECs) were incubated with **HAMA** and **HPD** hydrogels for 7 d. The corresponding chondrogenic, fibrogenic, and angiogenic evaluations were examined via immunofluorescent evaluation reported eleswhere.^[^ ^53^, ^54^, ^55^ ^]^ Col II expression of chondrocytes, *α*‐SMA expression of fibroblasts, and VEGF expression of HUVECs were evaluated.

### Gene Expression Analyses through RT‐PCR

After culturing the constructs for 1 week, total RNA of cells was isolated using TRIzol reagent (Life Technologies) following the manufacturer's protocol. RNA concentration was measured using a Nanodrop (Thermo Scientific). Reverse‐transcription was performed with a cDNA synthesis kit (Thermo Scientific) following manufacturer's instructions. Gene expressions were analyzed quantitatively with SYBR‐green using 7500 Real‐Time PCR system (Applied Biosystems, Life Technologies). The primers and probes for ACAN, SOX9, VEGF, CD31, *α*‐SMA, COL1A1, and *β*‐actin were designed based on published gene sequences (NCBI and PubMed). Expression level for each gene was normalized with *β*‐actin. Each sample was assessed in triplicate.

### Bioprinting of Trachea Analogues

Final gel precursors of **C** rings were composed of 10% w/v **GelMA**, 2% w/v **CSMA**, and 1% w/v **ACMMA** with 0.2% w/v LAP. Final gel precursors of **VF** rings were composed of 2% w/v **HAMA**, 5% w/v **8‐ARM‐PEG**, and 1% w/v **ADMMA** with 0.2% w/v LAP. For cell‐free printing, gel precursors were directly used or dyed with either methylene blue (**GCC** gel) or rhodamine B (**HPD** gel). The two gel precursors were loaded separately into 5 mL syringes equipped with 0.21‐mm‐diameter needles. The syringes were then mounted into the syringe pump extruder on a 3D BioArchitect work station (Regenovo). Temperatures of syringes and the platform were maintained at 16±1 °C. To fabricate one single layer of ring‐shaped construct (9.0 mm of external diameter, 6.0 mm of internal diameter, and 2.0 mm of height for **C** rings, 1.0 mm of height for **VF** rings), 13 layers of each bioink were printed and photocrosslinked upon light irradiation (365 nm, 20 mW cm^−2^) within 30 s. To print the trachea‐shaped construct, two types of rings were printed alternately. Then, light irradiation (365 nm, 20 mW cm^−2^) was applied during switching of extruders. Printing parameters were used as follows: line gap: 500 µm; layer thickness: 160 µm; photocrosslinking time: 30 s per ring; pneumatic pressure: 0.2 MPa for GCC bioinks and 0.075 MPa for HPD bioinks; extrusion speed: 6 mm s^−1^ for GCC bioinks and 12 mm s^−1^ for HPD bioinks.

For bioprinting, two type of cells (chondrocytes and fibroblasts) were mixed homogeneously in the bioinks at the last step. The **C** rings were bioprinted using ***p*‐CB** with 1 × 10^8^ mL^−1^ of chondrocytes and **VF** rings were bioprinted using ***p*‐VFB** with 5 × 10^7^ mL^−1^ of fibroblasts respectively according to previous researches.^[^ ^11^, ^26^ ^]^ The bioprinted constructs were incubated in Dulbecco's modified eagle medium (DMEM), supplemented with 10% fetal bovine serum (FBS) and 1% penicillin/streptomycin in a 37 °C and 5% CO_2_ immediately after bioprinting. Each step was strictly conducted under sterile conditions.

### Lightsheet Scanning of Bioprinted Trachea

For live cell imaging, chondrocytes were labeled with green fluorescent proteins (GFP; Abcam) and fibroblasts were labeled with red fluorescent proteins (RFP; Abcam) according to the manufacturer's instructions. Both types of cells were mixed with the bioinks at the corresponding cell density. A miniature trachea sample (2.5 × 2.5 × 2.0 mm^3^) was bioprinted and mounted in 1% low‐melt agarose in front of the detection lens in the sample chamber. Whole trachea fluorescence images were acquired by lightsheet fluorescence microscopy (LSFM) (Lightsheet Z.1, Zeiss). Raw image data were collected in a lossless 16‐bit TIFF format. 3D reconstruction images and videos were performed with Arivis Vision 4D and Imaris. Fluorescent relative intensity of alternant **CVFIT** was analyzed by the ImageJ software (National Institutes of Health). Green fluorescence of GFP‐labeled chondrocytes was normalized as positive values, while red fluorescence of RFP‐labeled fibroblasts was normalized as negative values.

### Immunofluorescence Staining of Cells

To demonstrate biological functions of bioprinted tissues, immunofluorescence staining of cell‐specific markers were performed. At designed time points of culture, the bioprinted tissues were treated with 0.1% w/v Triton X‐100 in PBS for 30 min to permeabilize the cell membrane. The samples were then blocked with 1% w/v BSA in PBS for 1 h at room temperature. F‐actin cytoskeleton was stained by incubating bioprinted constructs with Alex Fluor 594‐phalloidin (1:40 dilution in 0.1% w/v BSA) at room temperature for 1 h, followed by DAPI staining (1:200). For other antibody staining, the bioprinted tissues were incubated with primary antibodies (1:200) at 4 °C overnight. After washing with PBS, the samples were incubated with secondary antibodies (Alexa Fluor 594‐ or 488‐conjugated goat antimouse or goat antirabbit antibodies (1:200 dilution) at room temperature for 1 h. Samples were washed with PBS and then stained with DAPI for 5 min at room temperature. Finally, the samples were examined by CLSM (Leica, TCS SP8 STED3X).

### Trachea Regeneration in Subcutaneous Environment of Nude Mice

Male nude mice (5 week old) were divided into two groups: bioprinted **CVFIT** was implanted subcutaneously as the experimental group (*n* = 4); bioprinted cartilage tube was implanted subcutaneously as the control group (*n* = 4). For both groups, silicon tubes (6 mm external diameter) with similar length to trachea analogues were inserted as the internal mechanical support. After certain periods (2, 4, 6, and 8 weeks) of in vivo implantation, the silicon tubes were directly removed with forceps and the regenerated tissues were used for qualitative and quantitative evaluations.

### Trachea Reconstruction in Rabbits

New Zealand white rabbits (1‐month‐old) were used to evaluate the feasibility of in situ segmental trachea reconstruction with bioprinted **CVFIT** (*n* = 4). Briefly, the bioprinted **CVFIT** was stacked on a silicon tube, and wrapped with platysma muscle to facilitate trachea regeneration. After 8 weeks of in vivo implantation, the regenerated trachea with a bipedicle muscle flap were carefully dissected from the surrounding tissue to preserve the blood supply. Afterward, the silicon tube was also removed from the regenerated trachea using forceps. Then, the regenerated trachea was end‐to‐end anastomosed with the cut ends of the native trachea using 5–0 absorbable sutures. Penicillin was administered for 7 d to avoid infection. X‐ray scanning and tracheoscopy were performed before rabbits were euthanized at 8 weeks postoperation for further evaluations.

### Histological and Immunohistochemical Analyses

The samples were fixed in 4% paraformaldehyde, embedded in paraffin, and sectioned. Each section was stained with hematoxylin and eosin (H&E) and safranin‐O to evaluate its structure and cartilage and fibrous ECM deposition in the regenerated tissue. Expression of Col II was detected by immunohistochemical staining to further verify a cartilage‐specific phenotype. Masson's trichrome staining was used to observe the collagen fibers. To exhibit vascularization, the expression level of CD31 was detected by immunohistochemical staining. Immunohistochemical fluorescence staining of COL II (green), COL I (orange), CD31 (red), and cell nuclei (DAPI, blue) were used to reveal tissue‐specific ECM deposition.

### Biochemical Analyses

The samples were collected and minced to conduct cartilage‐related biochemical evaluations. GAG, and COL II contents were quantified using the dimethylmethylene blue assay (Sigma‐Aldrich) and enzyme‐linked immunosorbent assay, respectively.

### Mechanical Tests

Young's moduli of the samples (regenerated and native trachea segments) were measured using a biomechanical analyzer (Instron‐5542). Each sample was subjected to compression with a continuous planar unconfined strain rate of 1 mm min^−1^ until 80% of the maximal deformation was reached, and the Young's modulus was calculated based on the linear region of the stress‐strain curve. For tensile testing, the samples were pulled in tension at a rate of 10 mm min^−1^ until failure. The applied force at failure point was recorded as maximum tensile force that the tissue could withstand.

### Statistical Analyses

Data (*n* = 4) were expressed as the means ± standard deviations. A one‐way analysis of the variance was used to determine the statistical significance of the difference between groups using GraphPad Prism 7.00 software, and a *p*‐value < 0.05 was considered statistically significant.

## Conflict of Interest

The authors declare no conflict of interest.

## Supporting information

Supporting Information

Supplemental Video 1

Supplemental Video 2

Supplemental Video 3

Supplemental Video 4

Supplemental Video 5

Supplemental Video 6

## Data Availability

The data that support the findings of this study are available from the corresponding author upon reasonable request.

## References

[1] M. J. McPhail , J. R. Janus , D. G. Lott , BMJ 2020, 369, m718. 32349978 10.1136/bmj.m718

[2] A. Dhasmana , A. Singh , S. Rawal , J. Tissue Eng. Regener. Med. 2020, 14, 653. 10.1002/term.3019 32064791

[3] Y. Xu , L. Duan , Y. Li , Y. She , J. Zhu , G. Zhou , G. Jiang , Y. Yang , Adv. Funct. Mater. 2020, 30, 1910067.

[4] L. G. Griffith , G. Naughton , Science 2002, 295, 1009. 11834815 10.1126/science.1069210

[5] E. Cosgriff‐Hernandez , A. G. Mikos , Mater. Today 2008, 25, 2345. 10.1007/s11095-008-9666-4 18607694

[6] A. Khademhosseini , R. Langer , Nat. Protoc. 2016, 11, 1775. 27583639 10.1038/nprot.2016.123

[7] Y. Li , Y. Xiao , C. Liu , Chem. Rev. 2017, 117, 4376. 28221776 10.1021/acs.chemrev.6b00654

[8] X. Luo , Y. Liu , Z. Zhang , R. Tao , Y. Liu , A. He , Z. Yin , D. Li , W. Zhang , W. Liu , Y. Cao , G. Zhou , Biomaterials 2013, 34, 3336. 23380355 10.1016/j.biomaterials.2013.01.060

[9] D. Li , Z. Yin , Y. Liu , S. Feng , Y. Liu , F. Lu , Y. Xu , P. Min , M. Hou , K. Li , A. He , W. Zhang , W. Liu , Y. Zhang , G. Zhou , Y. Cao , Acta Biomater. 2019, 89, 206. 30867137 10.1016/j.actbio.2019.03.003

[10] J. H. Park , J. M. Hong , Y. M. Ju , J. W. Jung , H. Kang , S. J. Lee , J. J. Yoo , S. W. Kim , S. H. Kim , D. Cho , Biomaterials 2015, 62, 106. 26041482 10.1016/j.biomaterials.2015.05.008

[11] Y. Xu , Y. Guo , Y. Li , Y. Huo , Y. She , H. Li , Z. Jia , G. Jiang , G. Zhou , Z. You , L. Duan , Adv. Funct. Mater. 2020, 30, 2004276.

[12] Y. Xu , J. Dai , X. Zhu , R. Cao , N. Song , M. Liu , X. Liu , J. Zhu , F. Pan , L. Qin , G. Jiang , H. Wang , Y. Yang , Adv. Mater. 2022, 34, 2106755. 10.1002/adma.202106755 34741771

[13] B. Derby , Science 2012, 338, 921. 23161993 10.1126/science.1226340

[14] R. L. Truby , J. A. Lewis , Nature 2016, 540, 371. 27974748 10.1038/nature21003

[15] Y. S. Zhang , G. Haghiashtiani , T. Hübscher , D. J. Kelly , J. M. Lee , M. Lutolf , M. C. McAlpine , W. Y. Yeong , M. Zenobi‐Wong , J. Malda , Nat. Rev. Methods Primers 2021, 1, 75.

[16] H. Ravanbakhsh , V. Karamzadeh , G. Bao , L. Mongeau , D. Juncker , Y. S. Zhang , Adv. Mater. 2021, 33, 2104370. 10.1002/adma.202104730 PMC8971140 34596923

[17] X. Ma , X. Qu , W. Zhu , Y. Li , S. Yuan , H. Zhang , J. Liu , P. Wang , C. S. E. Lai , F. Zanella , G. Feng , F. Sheikh , S. Chien , S. Chen , Proc. Natl. Acad. Sci. USA 2016, 113, 2206. 26858399 10.1073/pnas.1524510113 PMC4776497

[18] L. Ouyang , C. B. Highley , W. Sun , J. A. Burdick , Adv. Mater. 2017, 29, 1604983. 10.1002/adma.201604983 27982464

[19] S. Lee , E. S. Sani , A. R. Spencer , Y. Guan , A. S. Weiss , N. Annabi , Adv. Mater. 2020, 32, 2003915. 10.1002/adma.202003915 PMC7658039 33000880

[20] M. Wang , W. Li , L. S. Mille , T. Ching , Z. Luo , G. Tang , C. E. Garciamendez , A. Lesha , M. Hashimoto , Y. S. Zhang , Adv. Mater. 2021, 2107038. 10.1002/adma.202107038 PMC8741743 34609032

[21] Y. S. Zhang , A. Khademhosseini , Science 2017, 356, eaaf3627. 28473537 10.1126/science.aaf3627 PMC5841082

[22] K. Zhang , Q. Feng , Z. Fang , L. Gu , L. Biao , Chem. Rev. 2021, 121, 11149. 34189903 10.1021/acs.chemrev.1c00071

[23] J. Thiele , Y. Ma , S. M. C. Bruekers , S. Ma , W. T. S. Huck , Adv. Mater. 2014, 26, 125. 24227691 10.1002/adma.201302958

[24] T. E. Brown , K. S. Anseth , Chem. Soc. Rev. 2017, 46, 6532. 28820527 10.1039/c7cs00445a PMC5662487

[25] W. Zhang , B. Bao , F. Jiang , Y. Zhang , R. Zhou , Y. Lu , S. Lin , Q. Lin , X. Jiang , L. Zhu , Adv. Mater. 2021, 2105667. 10.1002/adma.202105667 34605063

[26] Y. Hua , H. Xia , L. Jia , J. Zhao , D. Zhao , X. Yan , Y. Zhang , S. Tang , G. Zhou , L. Zhu , Q. Lin , Sci. Adv. 2021, 7, eabg0628. 34433558 10.1126/sciadv.abg0628 PMC8386926

[27] A. Lee , A. R. Hudson , D. J. Shiwarski , J. W. Tashman , T. J. Hinton , S. Yerneni , J. M. Bliley , P. G. Campbell , A. W. Feinberg , Science 2019, 365, 482. 31371612 10.1126/science.aav9051

[28] B. Grigoryan , S. J. Paulsen , D. C. Corbett , D. W. Sazer , C. L. Fortin , A. J. Zaita , P. T. Greenfield , N. J. Calafat , J. P. Gounley , A. H. Ta , F. Johnansson , A. Randles , J. E. Rosenkrantz , J. D. Louis‐Rosenberg , P. A. Galie , K. R. Stevens , J. S. Miller , Science 2019, 364, 458. 31048486 10.1126/science.aav9750 PMC7769170

[29] F. E. Freeman , P. Pitacco , L. A. V. Dommelen , J. Nulty , D. C. Browe , J. Y. Shin , E. Alsberg , A. D. J. Kelly , Sci. Adv. 2020, 6, eabb5093. 32851179 10.1126/sciadv.abb5093 PMC7428335

[30] W. W. Cho , B. S. Kim , M. Ahn , Y. H. Ryu , D. H. Ha , J. S. Kong , J. W. Rhie , D. W. Cho , Adv. Healthcare Mater. 2021, 10, 2001693. 10.1002/adhm.202001693 33236508

[31] B. S. Kim , M. Ahn , W. W. Cho , G. Gao , J. Jang , D. W. Cho , Biomaterials 2021, 272, 120776. 33798956 10.1016/j.biomaterials.2021.120776

[32] H. Kim , B. Kang , X. Cui , S. H. Lee , K. Lee , D. W. Cho , W. Hwang , T. B. F. Woodfield , K. S. Lim , J. Jang , Adv. Funct. Mater. 2021, 31, 2011252.

[33] S. H. Kim , Y. B. Seo , Y. K. Yeon , Y. J. Lee , H. S. Park , M. T. Sultan , J. M. Lee , J. S. Lee , O. J. Lee , H. Hong , H. Lee , O. Ajiteru , Y. J. Suh , S. Song , K. Lee , C. H. Park , Biomaterials 2020, 260, 120281. 32858503 10.1016/j.biomaterials.2020.120281

[34] J. H. Park , J. Y. Park , I. Nam , M. Ahn , J. Y. Lee , S. H. Choi , S. W. Kim , D. Cho , Biomaterials 2018, 185, 276. 30261427 10.1016/j.biomaterials.2018.09.031

[35] S. Kim , H. R. Lee , S. J. Yu , M. Han , D. Y. Lee , S. Y. Kim , H. Ahn , M. Han , T. Lee , T. Kim , S. K. Kwon , S. G. Im , N. S. Hwang , Proc. Natl. Acad. Sci. USA 2015, 112, 15426. 26621717 10.1073/pnas.1504745112 PMC4687567

[36] J. H. Park , M. Ahn , S. H. Park , H. Kim , M. Bae , W. Park , S. J. Hollister , S. W. Kim , D. Cho , Biomaterials 2021, 279, 121246. 34775331 10.1016/j.biomaterials.2021.121246 PMC8663475

[37] L. Zhang , L. Fu , X. Zhang , L. Chen , Q. Cai , X. Yang , Biomater. Sci. 2021, 9, 1547. 33439158 10.1039/d0bm01595d

[38] L. R. Lopes , A. F. Silva , O. S. Carneiro , Addit. Manuf. 2018, 23, 45.

[39] B. S. Kim , S. Das , J. Jang , D. W. Cho , Chem. Rev. 2020, 120, 10608. 32786425 10.1021/acs.chemrev.9b00808

[40] S. Krishtul , L. Baruch , M. Machluf , Adv. Funct. Mater. 2020, 30, 1900386.

[41] F. Pati , J. Jang , D. Ha , S. W. Kim , J. Rhie , J. Shim , D. Kim , D. Cho , Nat. Commun. 2014, 5, 3935. 24887553 10.1038/ncomms4935 PMC4059935

[42] M. Ali , A. Kumar PR , J. J. Yoo , F. Zahran , A. Atala , S. J. Lee , Adv. Healthcare Mater. 2019, 8, 1800992. 10.1002/adhm.201800992 PMC7039535 30725520

[43] W. Kim , H. Lee , J. Lee , A. Atala , J. J. Yoo , S. J. Lee , G. H. Kim , Biomaterials 2020, 230, 119632. 31761486 10.1016/j.biomaterials.2019.119632 PMC7141931

[44] D. O. Visscher , H. Lee , P. Zuijlen , M. N. Helder , A. Atala , J. J. Yoo , S. J. Lee , Acta Biomater. 2021, 121, 193. 33227486 10.1016/j.actbio.2020.11.029 PMC7855948

[45] K. Yue , X. Li , K. Schrobback , A. Sheikhi , N. Annabi , J. Leijten , W. Zhang , Y. S. Zhang , D. W. Hutmacher , T. J. Klein , A. Khademhosseini , Biomaterials 2017, 139, 163. 28618346 10.1016/j.biomaterials.2017.04.050 PMC5845859

[46] A. Abbadessa , M. M. Blokzijl , V. H. M. Mouser , P. Marica , J. Malda , W. E. Hennink , T. Vermonden , Carbohydr. Polym. 2016, 149, 163. 27261741 10.1016/j.carbpol.2016.04.080

[47] S. K. Seidlits , Z. Z. Khaing , R. R. Petersen , J. D. Nickels , J. E. Vanscoy , J. B. Shear , C. E. Schmidt , Biomaterials 2010, 31, 3930. 20171731 10.1016/j.biomaterials.2010.01.125

[48] J. Li , A. D. Celiz , J. Yang , Q. Yang , I. Wamala , W. Whyte , B. R. Seo , N. V. Vasilyev , J. J. Vlassak , Z. Suo , D. J. Mooney , Science 2017, 357, 378. 28751604 10.1126/science.aah6362 PMC5905340

[49] H. Yuk , C. E. Varela , C. S. Nabzdyk , X. Mao , R. F. Padera , E. T. Roche , X. Zhao , Nature 2019, 575, 169. 31666696 10.1038/s41586-019-1710-5

[50] M. Costantini , J. Idaszek , K. Szöke , J. Jaroszewicz , M. Dentini , A. Barbetta , J. E. Brinchmann , W. Święszkowski , Biofabrication 2016, 8, 035002. 27431574 10.1088/1758-5090/8/3/035002

[51] W. Liu , M. A. Heinrich , Y. Zhou , A. Akpek , N. Hu , X. Liu , X. Guan , Z. Zhong , X. Jin , A. Khademhosseini , Y. S. Zhang , Adv. Healthcare Mater. 2017, 6, 1601451. 10.1002/adhm.201601451 PMC5545786 28464555

[52] L. Jia , Y. Zhang , L. Yao , P. Zhang , Z. Ci , W. Zhang , C. Miao , X. Liang , A. He , Y. Liu , S. Tang , R. Zhang , X. Wang , Y. Cao , G. Zhou , Appl. Mater. Today 2020, 20, 100639.

[53] S. Varghese , N. S. Hwang , A. C. Canver , P. Theprungsirikul , D. W. Lin , J. Elisseeff , Matrix Biol. 2008, 27, 12. 17689060 10.1016/j.matbio.2007.07.002

[54] J. Y. Won , M. H. Lee , M. J. Kim , K. H. Min , G. Ahn , J. S. Han , S. Jin , W. S. Yun , J. H. Shim , Artif. Cells, Nanomed., Biotechnol. 2019, 47, 644. 30873886 10.1080/21691401.2019.1575842

[55] B. S. Kim , Y. W. Kwon , J. S. Kong , G. T. Park , G. Gao , W. Han , M. B. Kim , H. Lee , J. H. Kim , D. W. Cho , Biomaterials 2018, 168, 38. 29614431 10.1016/j.biomaterials.2018.03.040

