# Supplementary material for: Functional Trachea Reconstruction Using 3D‐Bioprinted Native‐Like Tissue Architecture Based on Designable Tissue‐Specific Bioinks
Source: Adv Sci (Weinh). 2022 Jul 26;9(29):2202181. doi: 10.1002/advs.202202181 (PMC9561786; doi:10.1002/advs.202202181)
Supplement: Supplementary file 1 — Supporting Information [file ADVS-9-2202181-s004.pdf]

ADVANCED SCIENCE
Open Access

Supporting Information

for *Adv. Sci.*, DOI 10.1002/advs.202202181

Functional Trachea Reconstruction Using 3D-Bioprinted Native-Like Tissue Architecture Based on Designable Tissue-Specific Bioinks

Yingying Huo, Yong Xu, Xiaodi Wu, Erji Gao, Anqi Zhan, Yujie Chen, Yixin Zhang, Yujie Hua, Wojciech Swieszkowski, Yu Shrike Zhang* and Guangdong Zhou**

Supporting Information

Functional Trachea Reconstruction Using 3D-Bioprinted Native-like Tissue Architecture Based on Designable Tissue-specific Bioinks

Yingying Huo, Yong Xu, Xiaodi Wu, Erji Gao, Anqi Zhan, Yujie Chen, Yixin Zhang, Yujie Hua, Wojciech Swieszkowski, Yu Shrike Zhang*, and Guangdong Zhou**

Y. Huo, Prof. Y. Zhang, Dr. Y. Hua, Prof. G. Zhou
Department of Plastic and Reconstructive Surgery, Shanghai 9th People's Hospital, Shanghai Jiao Tong University School of Medicine, Shanghai Key Laboratory of Tissue Engineering, Shanghai 200011, P. R. China.
National Tissue Engineering Center of China, Shanghai 200241, P. R. China.
E-mail: hyj137@shsmu.edu.cn; guangdongzhou@126.com

Prof. Y. S. Zhang
Division of Engineering in Medicine, Department of Medicine Brigham and Women's Hospital, Harvard Medical School, Cambridge, MA 02139, USA.
E-mail: yszhang@research.bwh.harvard.edu

Dr. Y. Xu, E. Gao
Department of Thoracic Surgery, Shanghai Pulmonary Hospital, School of Medicine, Tongji University, Shanghai 200433, P. R. China.

X. Wu, A. Zhan, Prof. G. Zhou
Research Institute of Plastic Surgery, Weifang Medical University, Weifang, Shandong 261053, P. R. China.

Prof. W. Swieszkowski
Materials Design Division, Faculty of Materials Science and Engineering, Warsaw University of Technology, Warsaw 02-507, Poland.

Dr. Y. Chen

Uli Schwarz Quantitative Biology Core Facility, Bio-Med Big Data Center, CAS Key Laboratory of Computational Biology, Shanghai Institute of Nutrition and Health, Chinese Academy of Sciences, Shanghai 200031, P. R. China.

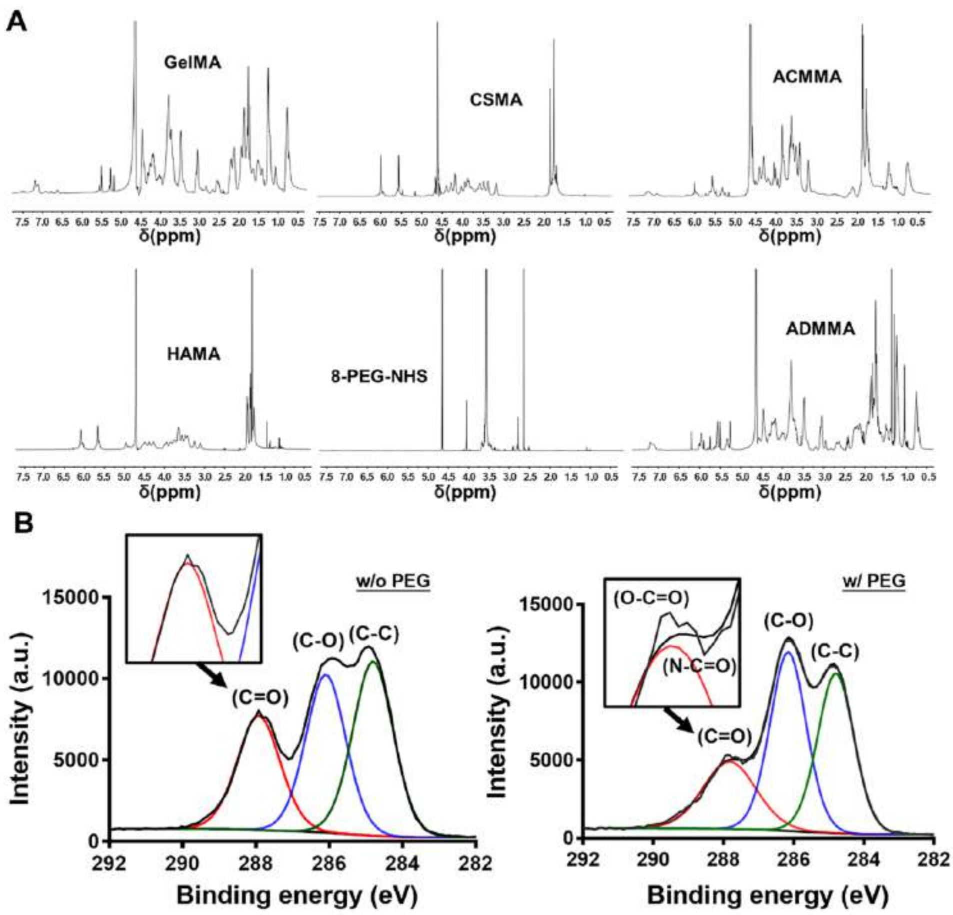

Figure S1. A) ^{1}H NMR spectra of methacryloyl-modified polymers of **GelMA**, **CSMA**, **ACMMA**, **HAMA**, and **ADMMA**, as well as **8-PEG-NHS**. B) XPS analyses of interfacial bonding with or without **8-PEG-NHS** treatment on **GelMA** gel surface.

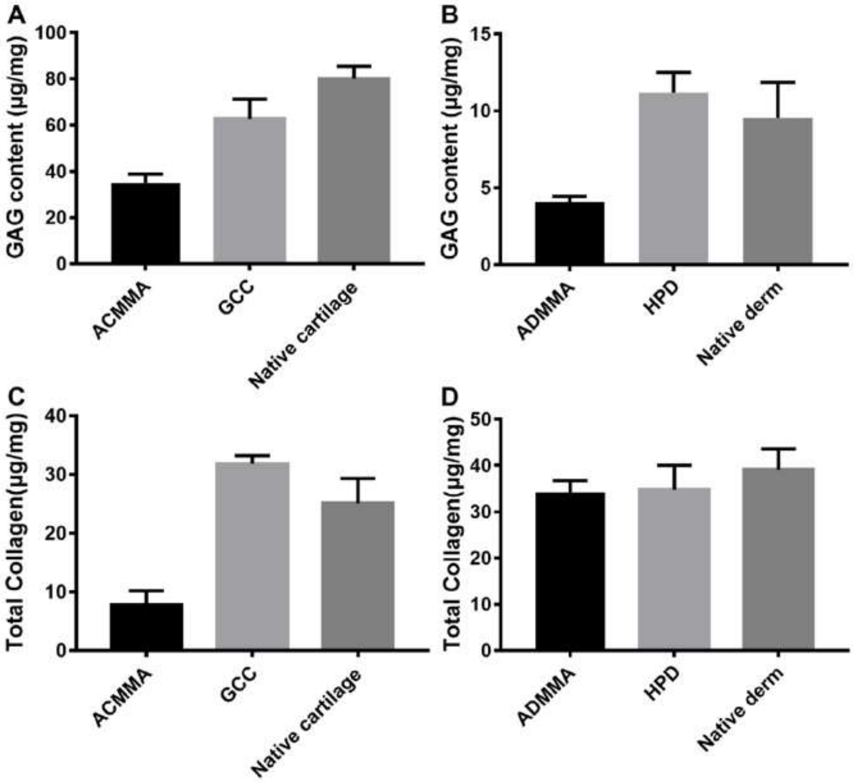

Figure S2. A-D) The GAG and collagen contents of **GCC** and **HPD** hydrogels compared to native cartilage and dermal tissue.

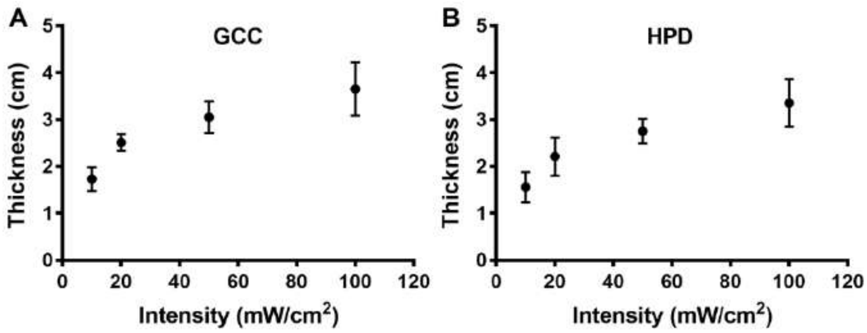

Figure S3. A-B) The relationships between 365-nm LED intensity (light irradiation time: 30 s) and photocuring thicknesses of **GCC** (A) and **HPD** (B) hydrogels.

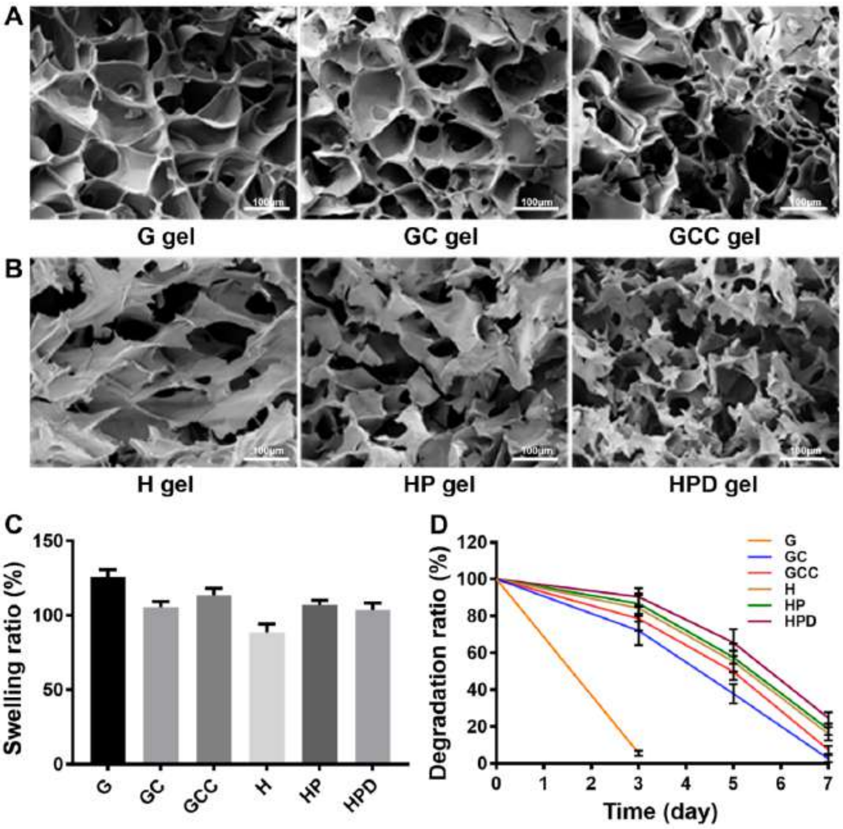

Figure S4. A, B) SEM images of **G**, **GC**, and **GCC** gels (A); **H**, **HP**, and **HPD** gels (B). C, D) Swelling (C) and degradation (D) behaviors of the hydrogels with different compositions.

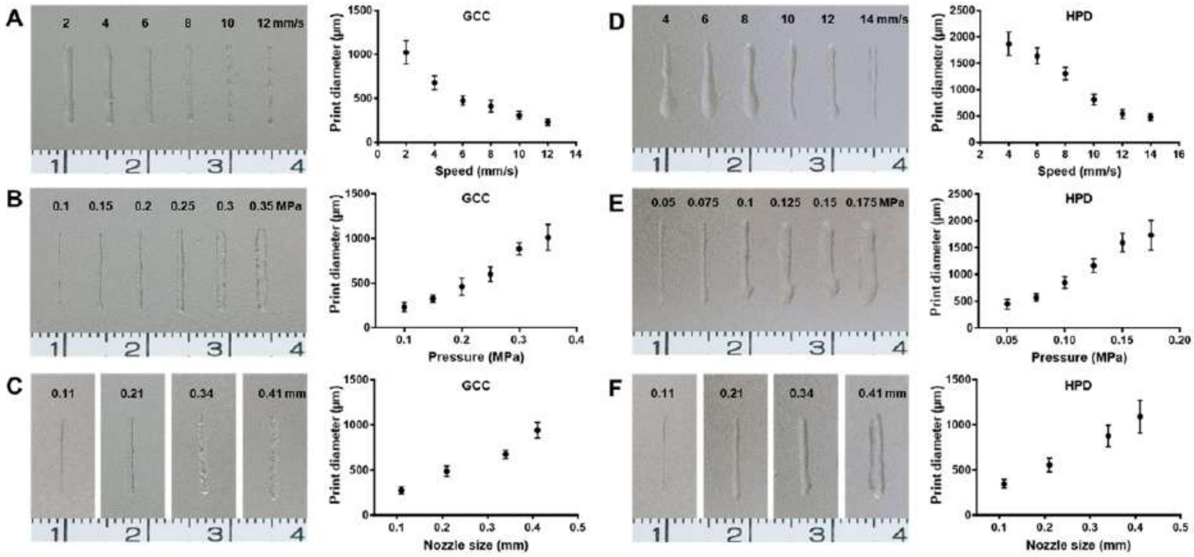

Figure S5. A-F) The printing parameters evaluation of **GCC** (A-C) and **HPD** (D-F) bioinks, such as printing speed, pressure, and nozzle size.

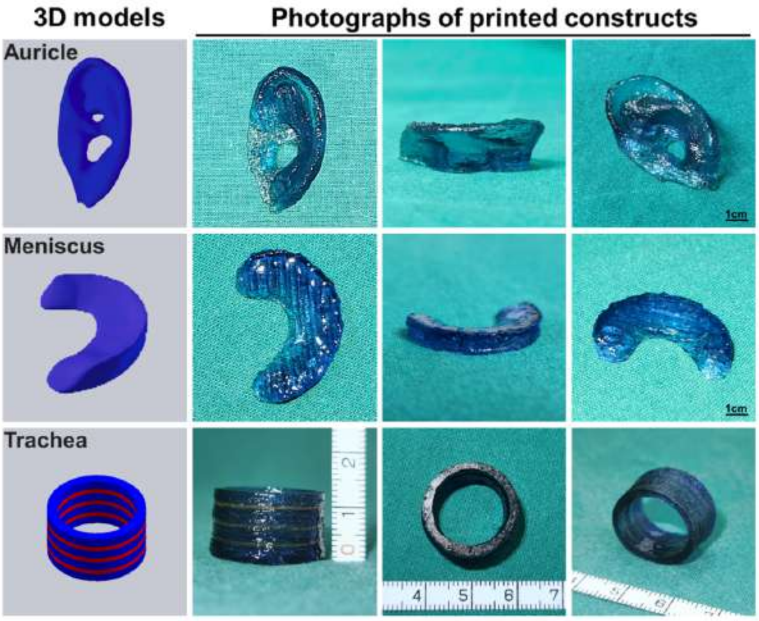

Figure S6. The complex 3D-printed tissue equivalents, such as auricle, meniscus, and trachea with real human tissue-matching sizes.

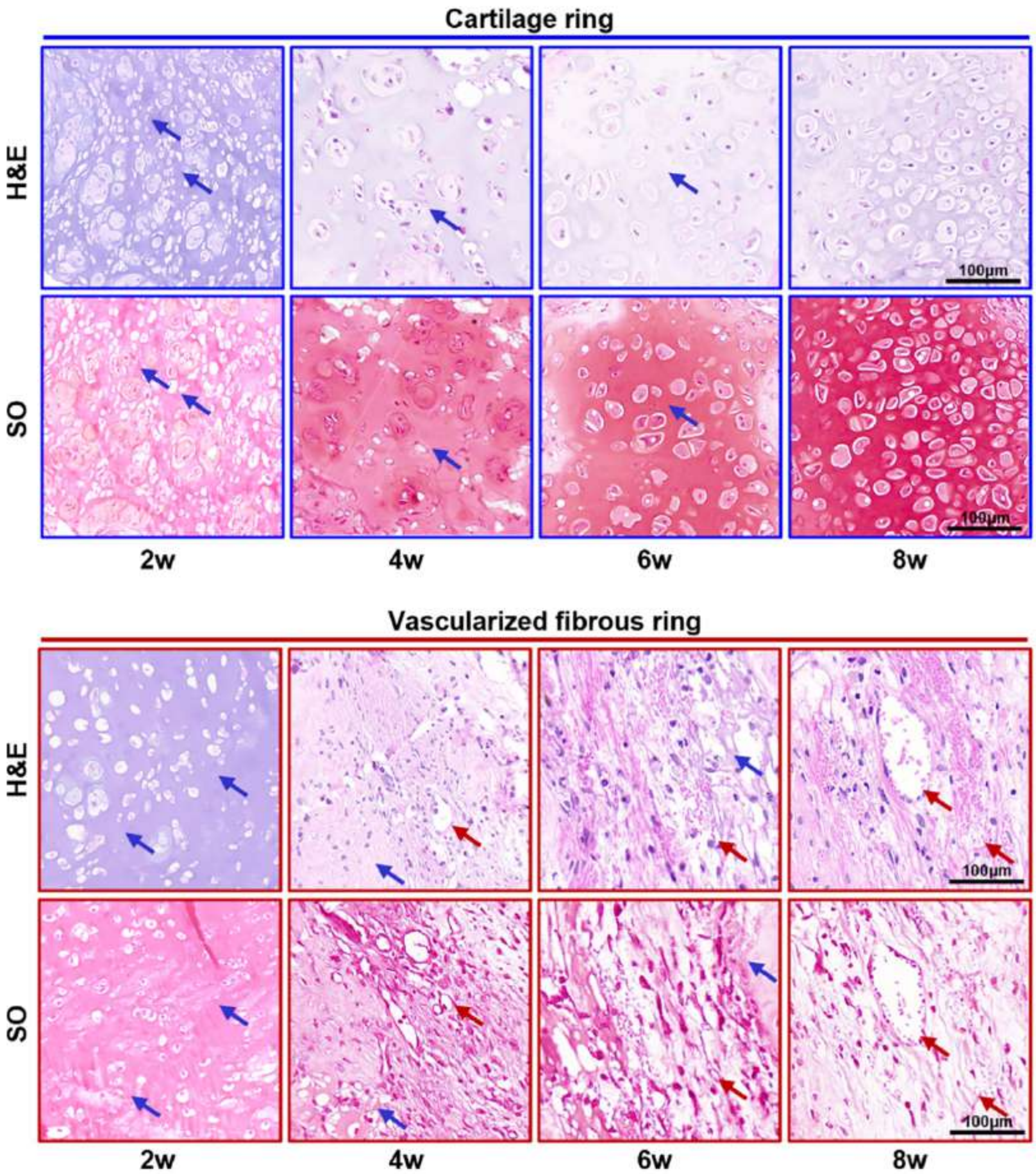

Figure S7. Histological examinations of H&E and safranin-O (SO) staining of the regenerated trachea showing the regions of cartilage and vascularized fibrous rings after 2, 4, 6, and 8 weeks post-implantation *in vivo*. Blue arrows represent the nondegraded hydrogels; red arrows represent the new blood vessels.

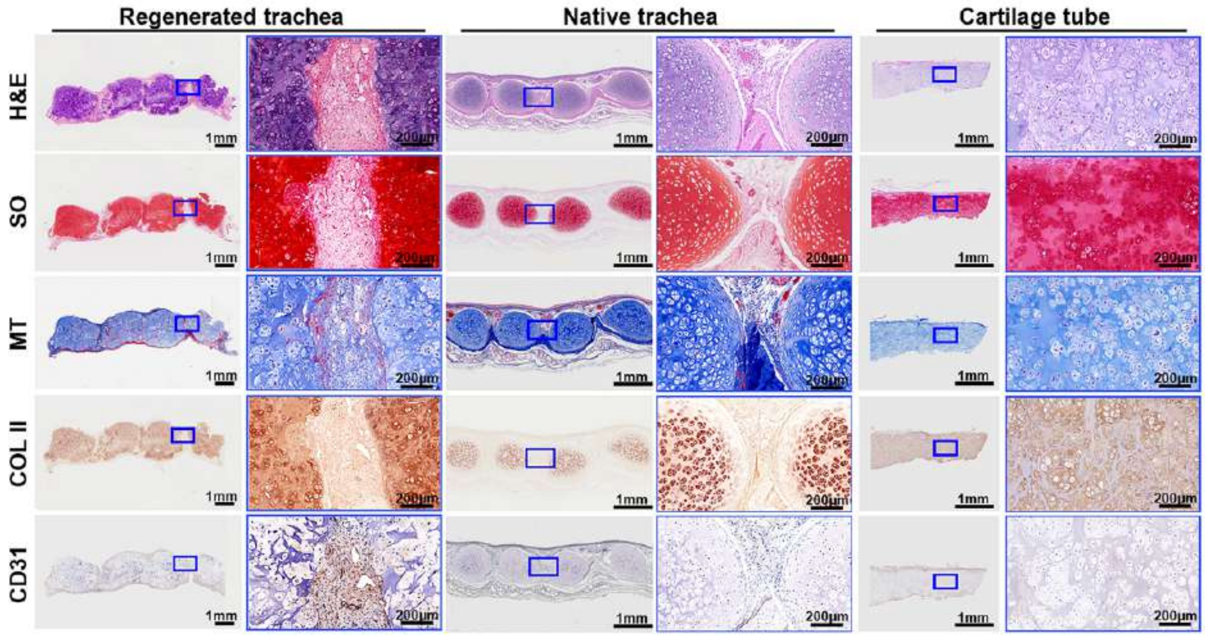

Figure S8. Histological examinations of H&E, safranin-O (SO), Masson's trichrome (MT), type II collagen (COL II), and CD31 staining (the longitudinal section) of the regenerated trachea, the native trachea, and the regenerated cartilage tube at 8 weeks post-implantation.

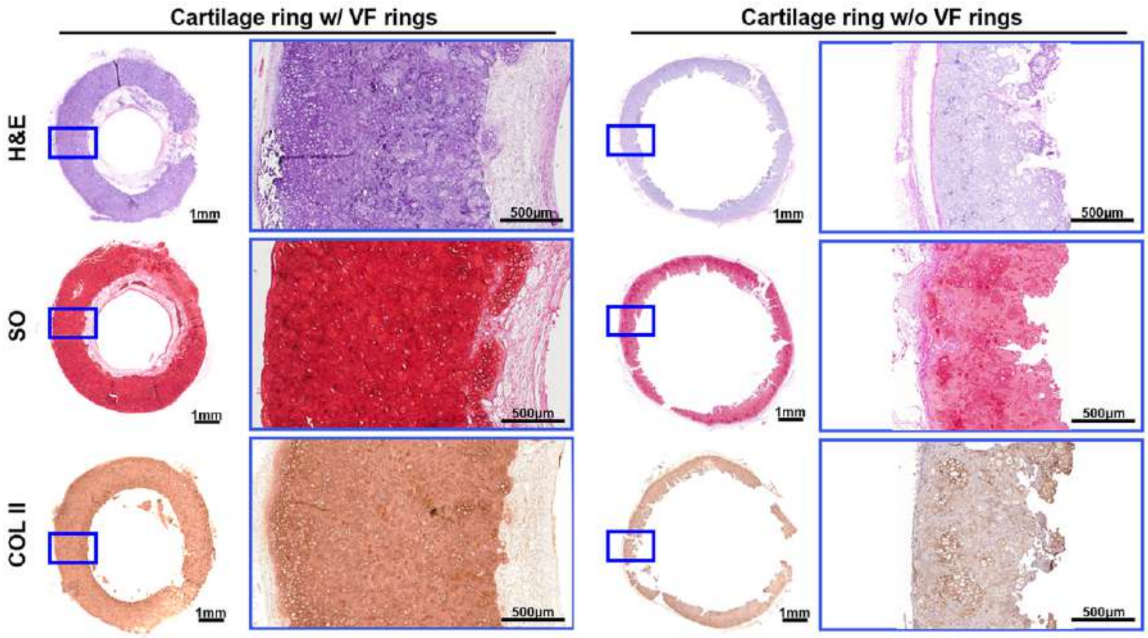

Figure S9. Histological examinations of H&E, safranin-O (SO), and type II collagen (COL II) staining (the transverse section) of the regenerated trachea and the cartilage tube at 8 weeks post-implantation.

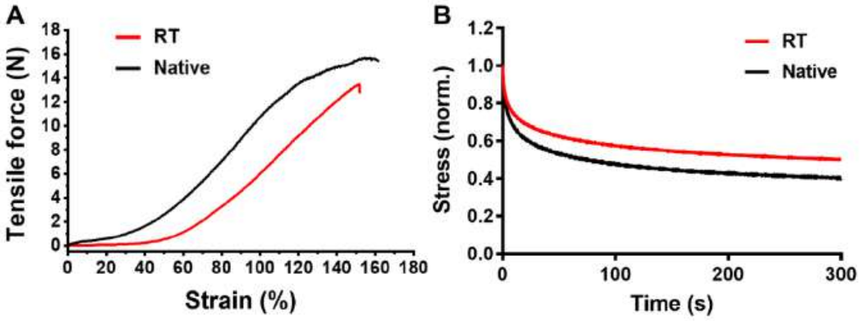

Figure S10. A-B) The nonlinear elastic properties of tensile (A) and stress relaxation (B) curves of the regenerated and native trachea. RT: regenerated trachea.

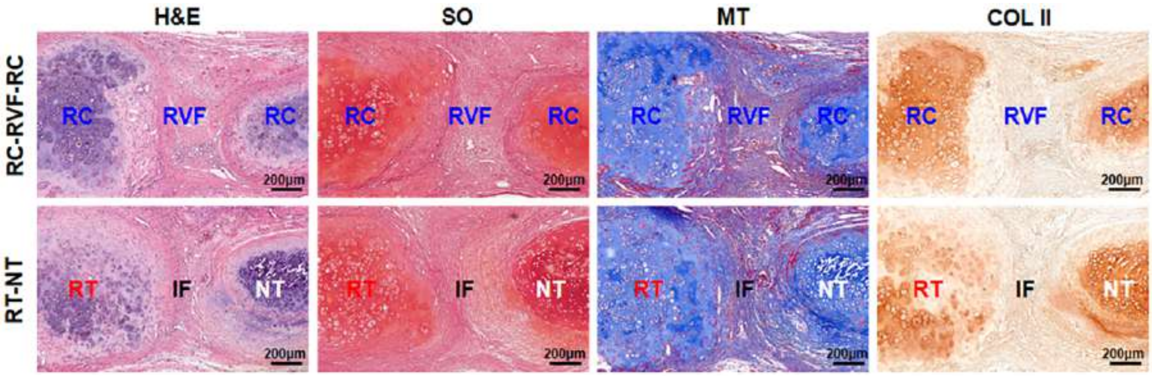

Figure S11. Interfacial histology of the regenerated cartilage-to-vascularized fibrous tissue-to-cartilage (RC-RVF-RC) region and the regenerated trachea-to-native trachea (RT-NT) region. RC: regenerated cartilage; RVF: regenerated vascularized fibrous tissue; RT: regenerated trachea. NT: native trachea. IF: interface.

Video S1. Video showing the photo-crosslinking processes of **GCC** and **HPD** hydrogels, as well as the interfacial integration between these two hydrogels.

Video S2. Video showing mechanical performance of the 3D-printed **CVFIT**.

Video S3. Video showing lightsheet images for visualizing cell distributions in the 3D-bioprinted **CVFIT**.

Video S4. Video showing compression-resistance of the regenerated 3D-bioprinted **CVFIT** at 4 and 8 weeks post-implantation *in vivo*.

Video S5. Video showing lateral stretchability of the regenerated 3D-bioprinted cartilage tube, regenerated 3D-bioprinted **CVFIT**, and the native trachea.

Video S6. Video showing the tracheoscopy examination of the *in situ* trachea reconstruction.
